# Identification and characterization of genes involving the early step of Juvenile Hormone pathway in *Helicoverpa armigera*

**DOI:** 10.1038/s41598-017-16319-z

**Published:** 2017-11-29

**Authors:** Wanna Zhang, Long Ma, Haijun Xiao, Chen Liu, Lin Chen, Shaolong Wu, Gemei Liang

**Affiliations:** 1 Institute of Entomology, Jiangxi Agricultural University, Nanchang, 330045 China; 2 State Key Laboratory for Biology of Plant Diseases and Insect Pests, Institute of Plant Protection, Chinese Academy of Agricultural Sciences, Beijing, 100193 China; 3 Jiangxi Key Laboratory of Bioprocess Engineering, Jiangxi Science & Technology Normal University, Nanchang, 330013 China; 4 China Tobacco Midsouth Agricultural Experimental Station, Changsha, 410128 China

## Abstract

Juvenile hormones (JHs) are crucial regulators for multiple physiological processes in insects. In the current study, 10 genes in mevalonate pathway involved in JH biosynthesis were identified from *Helicoverpa armigera*. Tissue-specific expression analysis showed that six genes were highly expressed in the head which contained the JH biosynthetic gland (corpora allata). Temporal expression pattern showed that 10 of 12 genes were highly transcribed in the late 2^nd^-instar when the *in vivo* JH titer reached the peak, indicating a tight correlation between JH titer and the transcription of JH synthetic pathway genes. Moreover, ingestion of methoprene, a JH analogue, significantly suppressed the transcription of nine JH biosynthetic genes and caused a feedback upregulation of the JH degradation enzyme. Particularly, the Acetoacetyl CoA thiolase (*HaAce*) and Farnesyl diphosphate synthase gene 4 (*HaFpps4*) showed high transcript abundance, and their temporal expressions keep pace with JH fluctuations. Further study by RNAi showed that knockdown of *HaFpps4* caused the decrease of JH titer, led to a negative effect on the transcript levels of other genes in JH pathway, and resulted in molting disturbance in larvae. Altogether, these results contribute to our understanding of JH biosynthesis in *H. armigera* and provide target genes for pest control based on JH-dependent regulation.

## Introduction

Juvenile hormones (JHs), synthesized and released by corpora allata (CA) which is located in the stomatogastric nervous system of insect brain^1^, are critical hormones involved in the regulation of insect molting and metamorphosis^2^. The molt and metamorphosis of insect larvae is preceded by the induction of JH action, in close cooperation with the regulation of ecdysteroid titers^3^. Normally, ecdysone causes larvae to molt to the next larval stage at the condition of JH being present, and it induces insect metamorphosis in absence of JH^4^. Recent studies revealed that JH is also involved in multiple other physiological processes during insect life cycle, including phase polyphenism, caste differentiation, diapause, reproduction and many other physiological functions^5–7^. Considering the extensive involvement of JH, a better knowledge of regulatory mechanisms that underlie the fluctuations of JH titers could help provide a potential method for insect control. Actually, JH analogues, such as methoprene, pyriproxyfen and fenoxycarb, have already been used in pest control, and the growth regulation of parasitic wasps^8,9^.

Change of JH titer in hemolymph is primarily regulated by the modulation in JH synthesis and catabolism through actions of specific enzymes^10^. The complete JH biosynthesis is accomplished by 13 separate enzymatic steps, divided into two metabolic sections (Fig. S1). The first section constitutes the mevalonate pathway, which begins from acetyl-CoA to farnesyl pyrophosphate (FPP) formation. In next section, FPP is finally converted to JH through the intermediates of Farnesal and Farnesoic acid^11^. The first section is conserved among invertebrates, while the next section is specific to insects. Intermediates in the mevalonate pathway are also needed for the biosynthesis of many important metabolites in plants and animals. In animals, the primary end-product of mevalonate pathway is cholesterol, and most research focuses on this pathway due to the underlying cause of cardiovascular disease by cholesterol. In insects, the peculiarity of mevalonate pathway is the capacity to synthesize JH due to the lack of relevant cholesterol synthesis enzymes. In insects, the mevalonate pathway involves eight enzymes and is divided into three steps, utilizing acetyl-CoA to form farnesyl diphosphate^12^. Firstly, enzymes of acetoacetyl CoA thiolase (*Ace*), HMG-CoA synthase (*Hmgs*), and HMG CoA reductase (*Hmgr*) are involved in catalyzing three units of acetyl-CoA into mevalonate. Then, mevalonate is converted to isopentenyl diphosphate (*Ipp*) through three enzymatic reactions catalyze by mevalonate kinase (*Mk*), phosphomevalonate kinase (*Pk*), and mevalonate diphosphate decarboxylase (*Dpp*)^13^. Finally, FPP synthase (*Fpps*) generates FPP by completing two sequential couplings: first IPP and dimethylallyl pyrophosphate (DMAPP) can condense in a head-to-tail manner to produce geranyl diphosphate (GPP); subsequently, GPP and IPP are condensed to yield FPP^12^. However, it has been difficult to identify the genes encoding enzymes from farnesyl diphosphate to JH because of the lack of vertebrate homologs.

Functional characterizations of enzymes in mevalonate pathway have been elucidated in many insects, such as *Bombyx mori*, *Aedes aegypti*, *Apis mellifera, Tribolium castaneum* and *Danaus plexippus*. In *B. mori*, it was found that one type of enzyme in each step of the mevalonate pathway was encoded by a single gene, except for Fpps^14^. Moreover, most mevalonate enzyme-encoding genes were highly enriched in the CA and their expression patterns correlated with the fluctuation of JH titer^14,15^. Transcription changes in mevalonate enzyme-encoding genes resulted in an increase or decrease in JH content, indicating a relevant role of these genes in JH biosynthesis. Until recently, enzymes involved in the early steps of JH pathway have been identified in vertebrates and limited in model insect species, and the functional studies are confined to Hmgs^16^, Hmgr^17,18^, Ippi^19,20^ and Fpps^21–25^. However, in agricultural pests, the identification and functional characterization of the mevalonate pathway enzyme encoding genes are poorly understood.

Besides of the involvement in JH biosynthesis, the mevalonate pathway also participates in other processes. For example, the pine engraver beetle *de novo* produces the monoterpenoid pheromone component ipsdienol via the mevalonate pathway^26^, and the mevalonate enzyme-encoding genes are proposed to be involved in the pheromone production in *Lutzomyia longipalpis* ^27^. Additionally, (E)-*ß*-farnesene, the key component of aphid alarm pheromone components^28^, iridoid, a defensive secretion in leaf-beetle larvae^29^, and farnesene, the termite defence secretion^30^, are all *de novo* synthesized through the mevalonate pathway in insects.

The cotton bollworm, *Helicoverpa armigera*, is one serious pest of cotton, corn, vegetables, and many other crops. Recently, transgenic cotton expressing the Cry-1 Ac gene from *Bacillus thuringiensis* (Bt) has suppressed this pest effectively^31^. However, field-evolved resistance to Cry1 Ac showed a significant increase in northern China^32^, which has resulted in an urgency to develop novel pest management strategies. The molting process of *H. armigera* is regulated by JH and 20-hydroxyecdysone, and studies revealed that silencing the genes involved in JH pathway blocked the normal metamorphosis process^33,34^. Obviously, deep insight into the enzyme-regulating mechanisms in JH biosynthetic pathway will provide several promising targets for pest management. In the current study, we identified 10 enzyme-encoding genes in the early steps of JH biosynthetic pathway, and quantified the tissue distribution and developmental profiles of these genes during insect life cycle. Finally, we functionally characterized the silencing effect of *HaFpps4* and *HaAce* on the JH biosynthesis and the response of other genes. This study broadens the knowledge about enzyme-encoding genes involved in the JH pathway in *H. armigera* and provides target genes for pest control based on JH-dependent regulation.

## Results

### Identification of genes encoding mevalonate pathway enzymes

**Figure 1:**
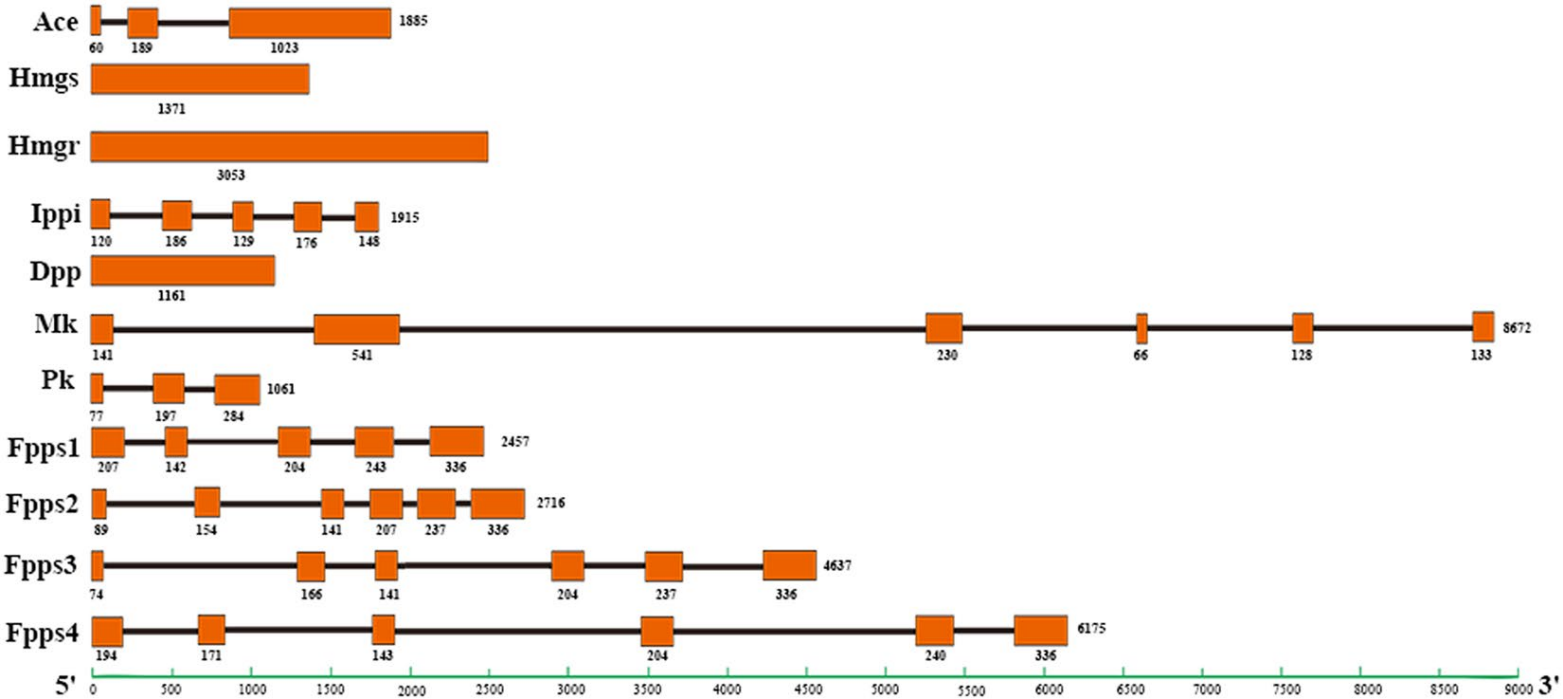
Genomic structure of 11 mevalonate enzymes-encoding genes in *H. armigera*. The saffron rectangles and straight-lines represent the exons and introns, respectively. Nucleotide numbers at the end of the diagram are indicated to provide a general estimation of gene size. The respective length of each exon is labeled under the rectangle.

The mevalonate pathway involved in Juvenile hormone biosynthesis is consisted of eight enzyme reactions. In our study, 10 enzyme-encoding genes were identified: *HaAce*, *HaHmgs*, *HaMk*, *HaPk*, *HaDpp*, *HaIppi*, *HaFpps*1*, HaFpps*2*, HaFpps*3 *and HaFpps*4 (Table S1). And the gene sequences were listed in Data Set S1. The predicted genomic structures of these genes, as well as *Hmgr* ^35^, were presented in Fig. 1. The genomic structures revealed that no introns were contained in *Hmgs*, *Hmgr* and *Dpp*, while introns which interrupt coding sequences occurred in the rest of genes (Fig. 1).

Fpps, a key enzyme in isoprenoid biosynthesis, catalyzes the final reaction of the mevalonate pathway to generate the precursor of JH. In this study, four transcripts encoding Fpps gene were identified in *H. armigera*. Sequence analysis revealed that the Fpps family contained several critical domains characteristic for isoprenyl diphosphate synthases, including two aspartate rich motifs named FARM (first aspartic rich motif) and SARM (second aspartic rich motif). However, the SARM domain of HaFpps1 was slightly different from other Fpps, containing a QNDXXD instead of QDDXXD motif (Fig. S2). Fpps proteins from 15 insects were used to construct rooted, neighbor-joining phylogenic trees, which showed that HaFpps1 was distinct from other sequences. Additionally, HaFpps2 was clustered with HaFpps3, but they were in distinct phylogenetic clusters from HaFpps4 (Fig. S2). The genomic structure of HaFpps1 gene was different from that of the other three genes, indicating that five exons were present in *HaFpps*1, while six were present in *HaFpps*2, *HaFpps*3 and *HaFpps*4 (Fig. 1).

### Tissue expression of JH biosynthetic pathway genes in *H. armigera*

**Figure 2:**
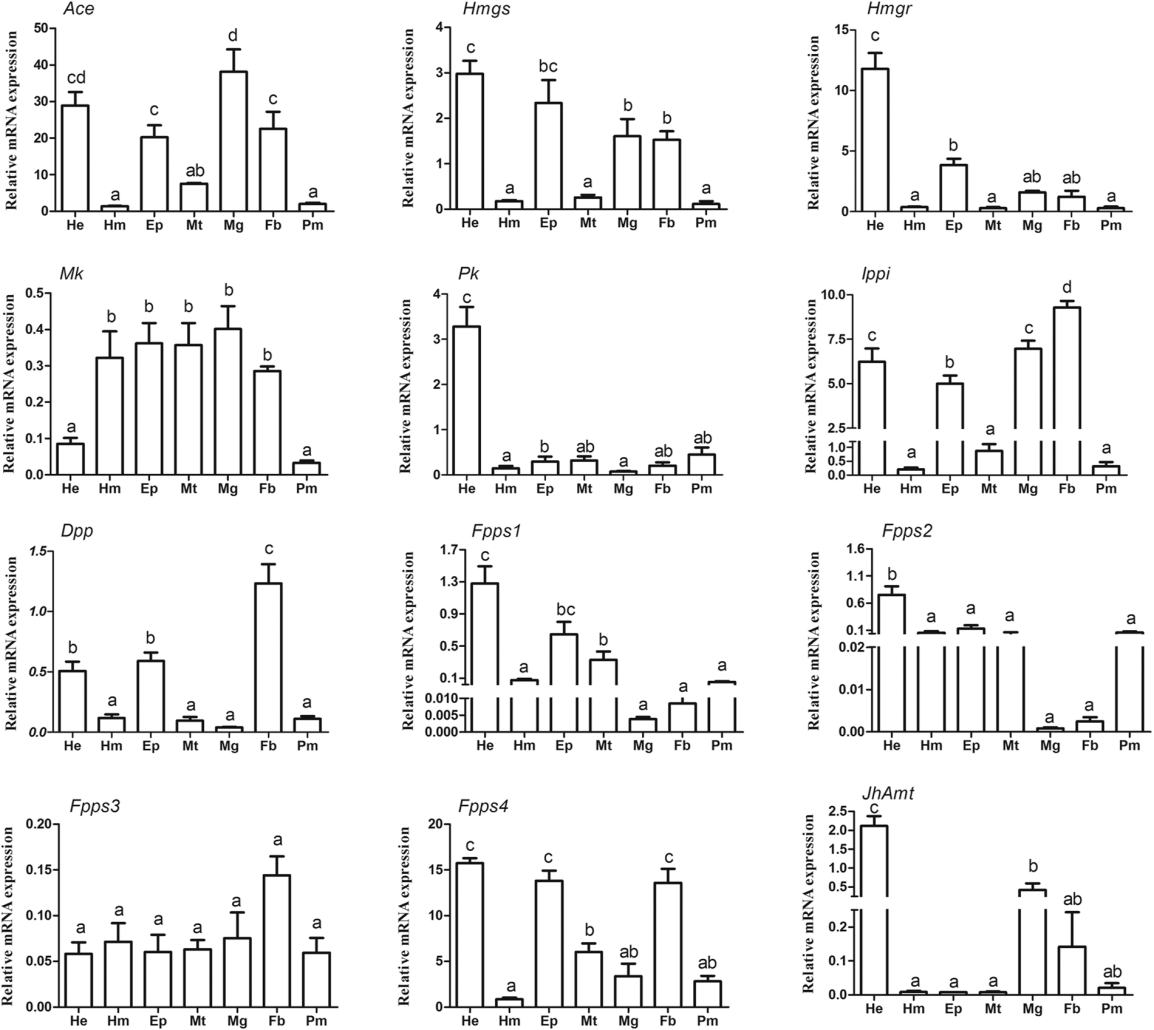
Tissue-specific transcript distribution of 11 mevalonate enzyme-encoding genes and *HaJhAmt* in *H. armigera*, 5th-instar 0 d larvae. He: head; Ep: epidermis; Fb: fat body; Mg: midgut; Mt: malpighian tubules; Hm: hemolymph; Pm: peritrophic matrix. The bars represent the average (±SE) of biological repeats. Different letters indicate a significant difference between specimens (P < 0.05).

The expression profiles of ten identified mevalonate enzyme-encoding genes were investigated, in addition to two relevant genes reported by other literature, *JhAmt* (JH acid methyltransferase) and *Hmgr* ^35^. Results showed that the transcription levels of these genes were consistent with their enzymatic activities in JH biosynthesis (Fig. 2). The expressions of seven genes (*HaAce*, *HaHmgs*, *HaHmgr*, *HaPk*, *HaFpps1*, *HaFpps2* and *JhAmt*) were highly enriched in head where the JH synthetic gland is located. Additionally, *HaMk, HaIppi, HaFpps*3 and *HaFpps*4 showed wide tissue distribution. Interestingly, *HaDpp* was widely distributed in multiple tissues, but was significantly higher in fat body (P < 0.05). In comparison of the transcript abundance of *Fpps* genes in single tissue, *Fpps*4 was significantly higher expressed than the other *Fpps* genes (Fig. S3).

### Expression profiles during developmental stages

**Figure 3:**
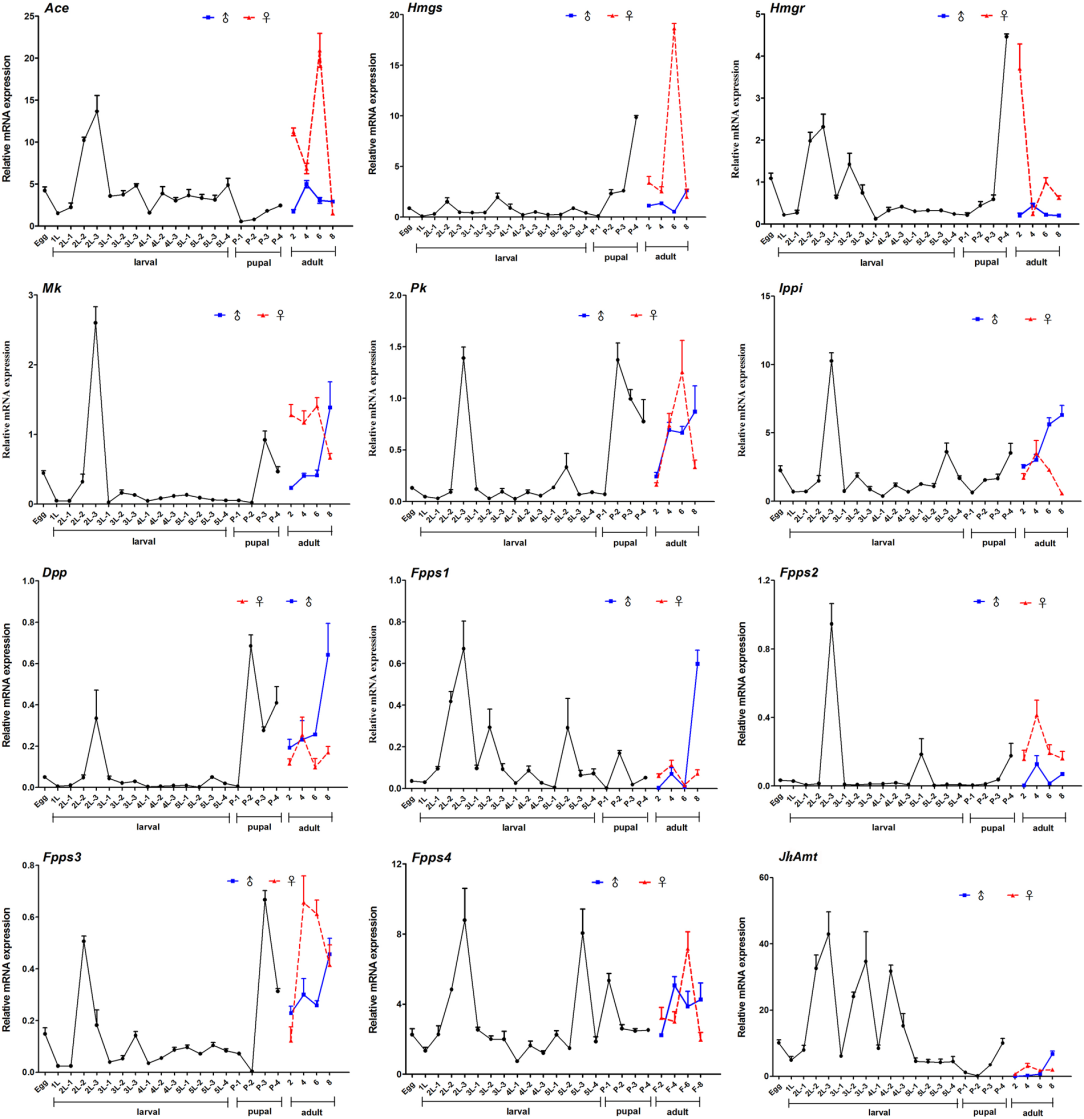
Expression patterns of 11 mevalonate enzyme-encoding genes as well as *JhAmt* in *H. armigera* at different developmental stages. Samples were prepared at an interval of one day during larval stages (1st-, 2nd-, 3rd-, 4th-, and 5th- instar) and two days for adult. Four types of pupae were prepared at the pupal stage (white pupae, green pupae, brown pupae and black pupae). For each sample, three independent pools of 3–50 individuals are measured. The bar represents the average (±SE) of the biological repeats.

The temporal expressions of genes in the mevalonate pathway were shown in Fig. 3. Overall, the transcription levels of mevalonate enzyme-encoding genes were sharply increased in the late 2^nd^-instar larval (the 3^rd^ day of 2^nd^-instar, namely 2L-3), showing a transient activation of these enzymes just before the molting of 2^nd^-instar larvae. And these transcriptions were significantly declined during the 4^th^- and 5^th^-instar. Moreover, an increase in transcription was observed in most mevalonate pathway genes at the late pupal stage, except for that of *HaAce*, *HaFpps1* and *HaFpps4*. It was also found that the expression levels of several enzyme-encoding genes in JH biosynthesis, such as *HaHmgs*, *HaMk*, *HaPk*, *HaDpp* and *HaFpps3*, were much higher in pupae and adults, relative to those in 3^rd^ to 5^th^ instar larvae.

### Dynamic fluctuations of JH titers during developmental stages

**Figure 4:**
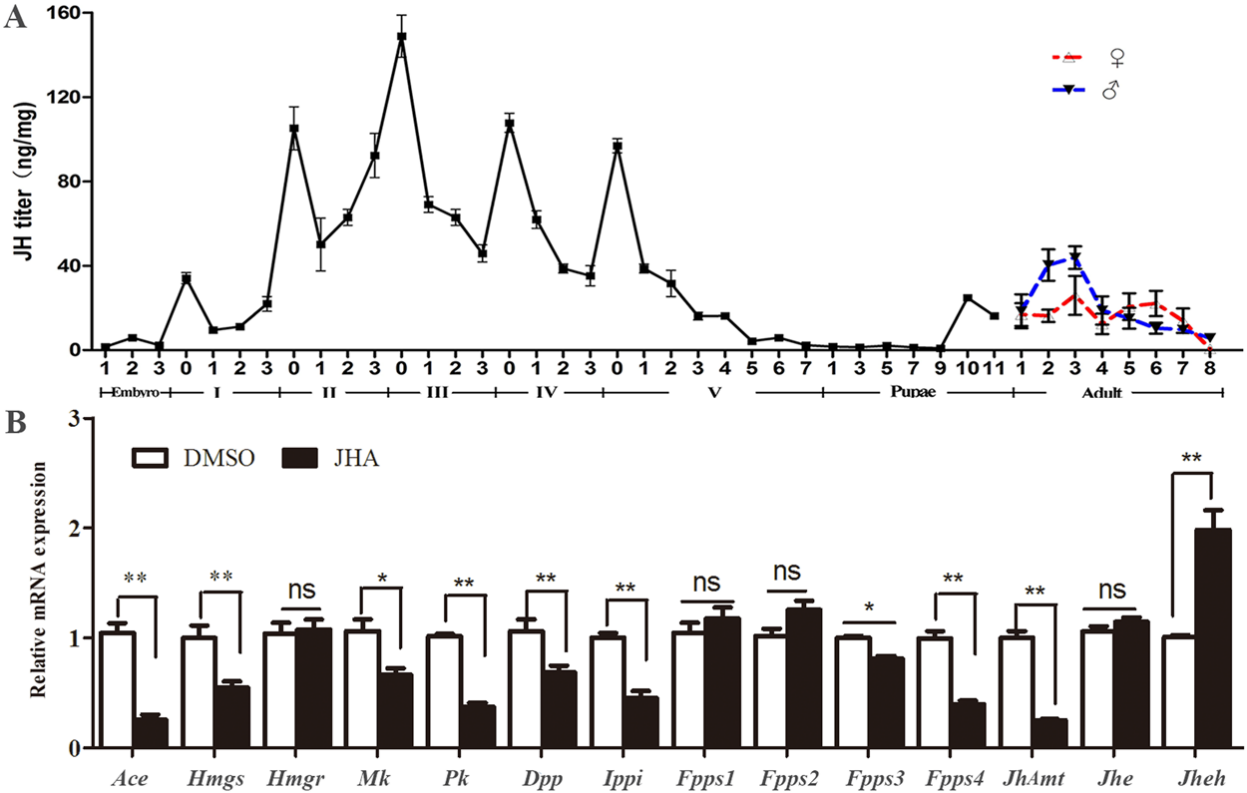
(**A**) The changes of JH III content *in vivo* of *H. armigera* from embryo to adult stages. Samples were collected daily except for a two-day collection in the pupal stage. (**B**) Effects of JHA ingestion on transcript levels of genes in mevalonate pathway. The standard error is represented by the error bar. Asterisks indicate statistically significant differences between groups (Student’s t-test): *P< 0.05, **P< 0.01. ns: no significant difference.

The temporal changes of JH titer during *H*. *armigera* development was depicted in Fig. 4A. Results showed that the JH biosynthesis remained active in larval stages, but declined at the last larval instar. It was especially interesting that a transient increase of JH titer was observed immediately preceding the molting of each larval instar. The highest JH titer occurred on the 3^rd^ day of 2^nd^-instar, which correlated well with the high transcriptions of enzymes in mevalonate pathway at the same period (Fig. 3). However, in the prepupal and pupal stages, rare amount of JH was produced. Subsequently, a sharp increase in JH content was observed at late pupal stage. After the emergence of adults, JH synthesis became active again and JH levels peaked at 3^rd^-day-old adults. Overall, the JH biosynthesis was more active in larval stages compared to that in pupae and adults.

### Effects of JHA ingestion on transcript levels of genes in mevalonate pathway

We examined the effect of JHA on expression levels of genes associated with JH biosynthesis (Fig. 4B). After treatment with 2 µg methoprene, the expressions of *HaAce*, *HaHmgs*, *HaMk*, *HaPk*, *HaIppi*, *HaDpp*, *HaFpps*3, *HaFpps*4 and *JhAmt* were significantly downregulated, while no significant differences were found in the expression level of *HaHmgr*, *HaFpps*1, *HaFpps*2 and Juvenile Hormone Esterase (*HaJhe*). Jhe is a crucial carboxylesterase for JH degradation in hemolymph. And the expression of *HaJheh* (Juvenile Hormone Epoxide Hydrolase), playing a pivotal role in JH catabolism in collaboration with Jhe, was induced after methoprene treatment (Fig. 4). Results indicated that JHA significantly suppressed the gene expression in JH biosynthesis, while induced the gene expressions in JH catabolic pathway.

### Knockdown of *HaFpps*4 suppressed the genes expression in mevalonate pathway

**Figure 5:**
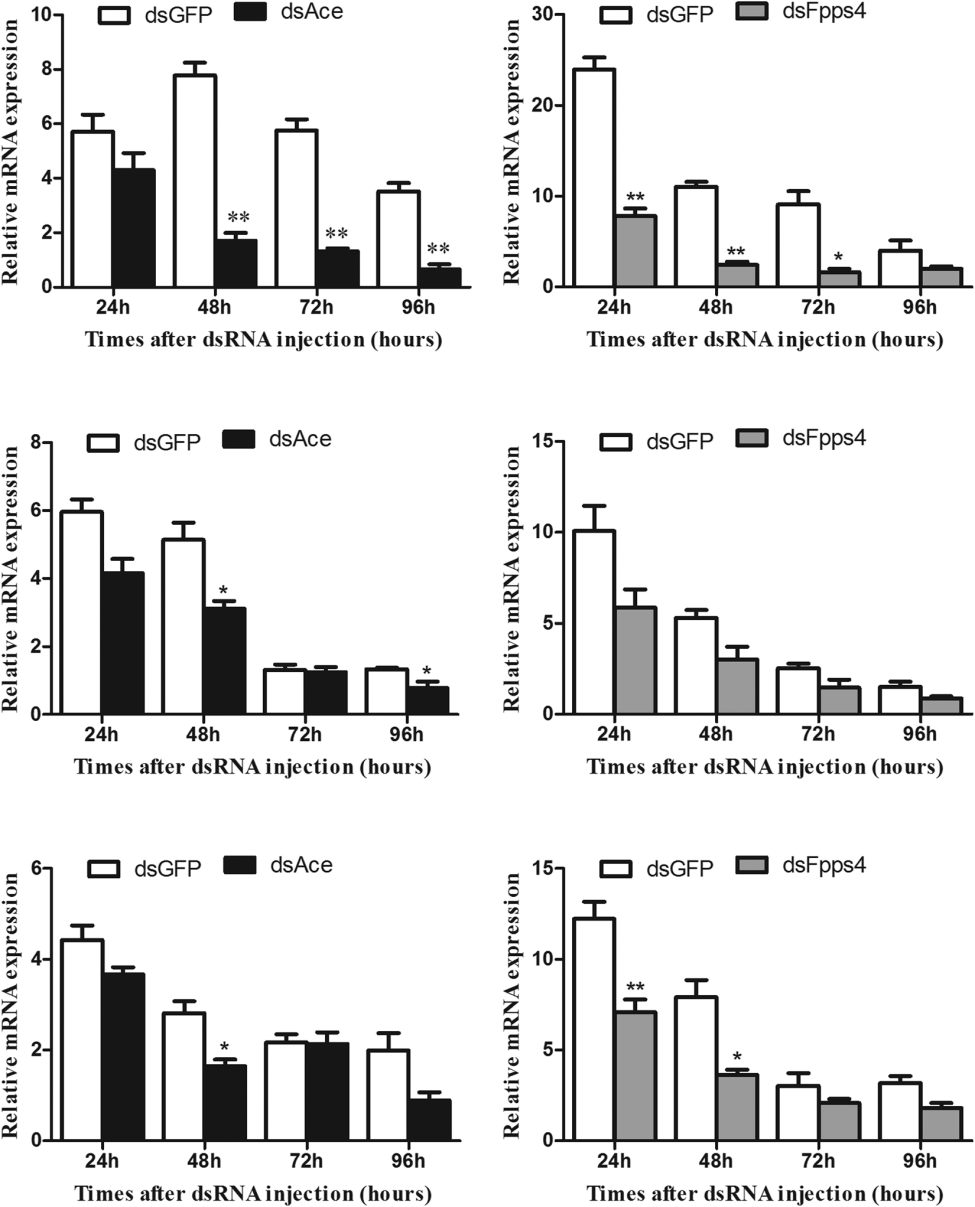
Detection of mRNA levels after dsRNA treatment in the newly molted fourth-instar larvae. *Ace* RNAi efficiency in the head (**A**), midgut (**C**) and epidermis (**E**) from 24 to 96 hours after dsRNA injection. *Fpps4* RNAi efficiency in the head (**B**), midgut (**D**) and epidermis (**F**) from 24 to 96 hours after dsRNA injection. Asterisks indicate statistically significant differences between groups (t-test): *P < 0.05, **P < 0.01.

As shown in Figs 2, 3 and 4, *HaAce* and *HaFpps*4 had high abundance in all tissues, and their peak transcriptions was detected immediately preceding the arrival of the highest JH titer in larval stage, indicating that *HaAce* and *HaFpps*4 may play important roles in early step of JH biosynthesis. Therefore, these two genes were selected for in-depth study using RNAi. Results showed that the dsRNA injections significantly decreased the expression levels of target *HaAce* and *HaFpps*4 in the tissue of head. Compared to the control group, the expression of *HaAce* was reduced by 78.12%, 77.02% and 81.53% at 48 h, 72 h and 96 h post injection, respectively (Fig. 5A). Similarly, the expression level of *HaFpps4* was reduced by 67.35%, 77.92%, 81.82% and 50.05% at 24 h, 48 h, 72 h and 96 h after dsRNA injection, respectively (Fig. 5B). However, no significant efficacy was detected in the tissue of midgut either in *dsAce* or *dsFpps4* treatment compared to the control group (Fig. 5C,D). In epidermis, the *HaFpps4* expression was significantly reduced at 24 h and 48 h post injection, but the silencing efficacy of *HaAce* lasted only 48 h after dsRNA injection (Fig. 5E,F).

**Figure 6:**
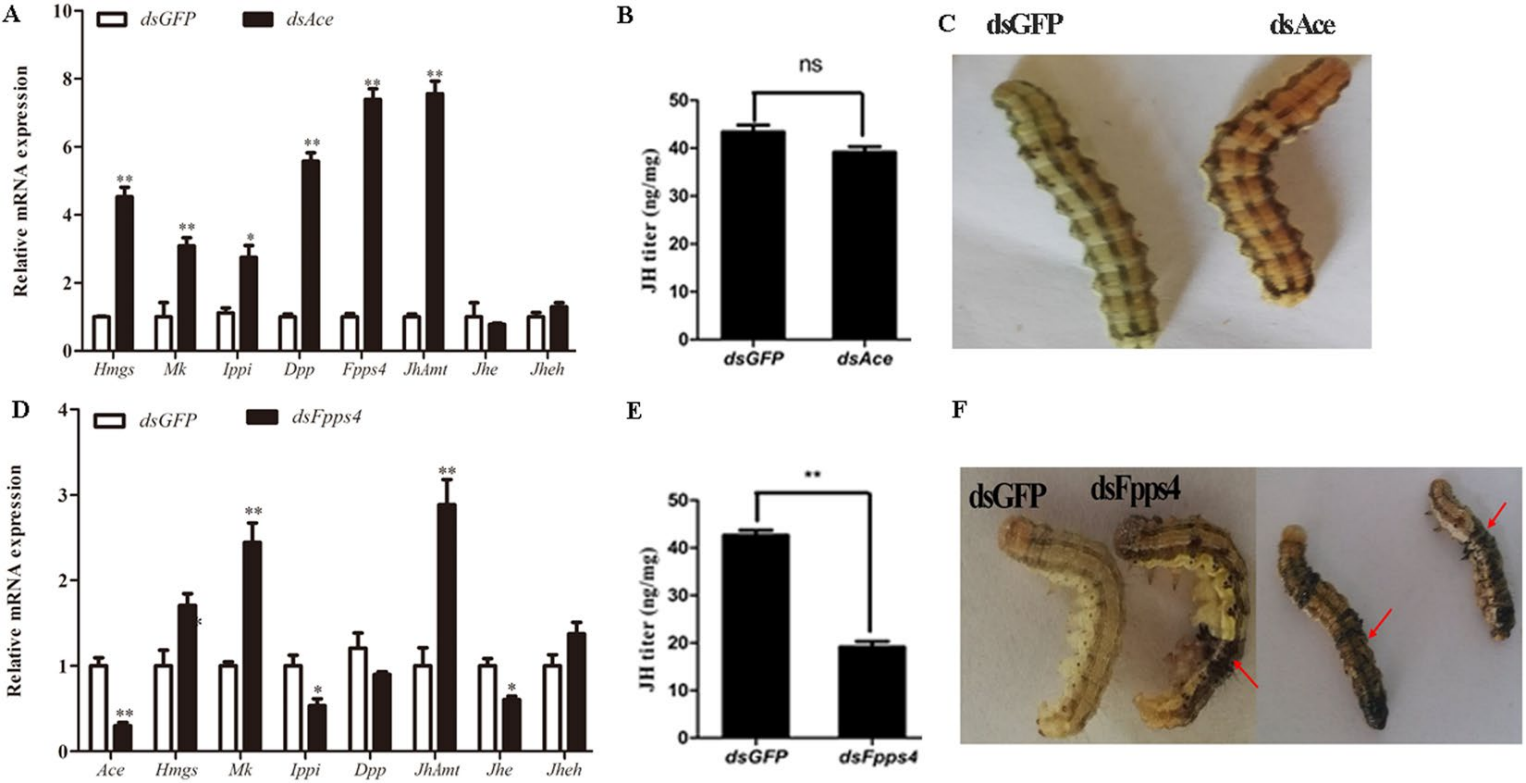
The effects of HaAce RNAi and HaFpps4 RNAi on the larvae. At 1d fourth-instar, larvae were microinjected with dsRNA of HaFpps4 (dsFpps4), dsRNA of HaAce (dsAce) or dsRNA of Green fluorescent protein (dsGFP) (control). The expression levels of the mevalonate pathway genes (**A**,**D**), the changes of JH titer (**B**,**E**) and the larvae development (**C**,**F**) were analyzed. The abnormal molting observed in dsFpps4 injection was labeled with red arrow. The standard error is represented by the error bar. Asterisks indicate statistically significant differences between groups (t-test): *P < 0.05, **P < 0.01, ns: no significant difference.

Additionally, knockdown of *HaAce* resulted in the up-regulation of *HaHmgs*, *HaMk*, *HaIppi*, *HaDpp*, *HaFpps4* and *JhAmt*, but no significant change was detected on the JH titer (Fig. 6A,B). When Fpps4 was silenced, the expressions of *HaAce*, *HaIppi* and *HaJhe* were significantly down-regulated, while *JhAmt* and *HaMk* were up-regulated, and there was no effect on the expression of *HaHmgs*, *HaDpp* and *HaJheh* (Fig. 6D). In contrast to *HaAce* silencing, the knockdown of *HaFpps*4 resulted in a decrease of JH titer *in vivo* relative to the control (Fig. 6E). Moreover, approximately 40% (n = 72) of the HaFpps4 RNAi larvae did not molt normally (Fig. 6F), while no abnormal molting was detected in the dsHaAce treatment group. The subcellular localization of HaFpps4 protein was investigated in Hi5 cell lines. Fluorescence microscopic observations revealed that HaFpps4 was dispersed in both cytoplasm and cell nucleus (Fig. S4).

## Discussion

Juvenile hormones play central roles in regulating many aspects of physiological processes in insects. Changes of JH titers are primarily regulated by modulations in the synthesis of JH, but also by JH catabolism through the action of specific enzymes^1^. To understand the regulation mechanisms of JH biosynthesis, it is important to study the enzymes involved in each step of JH biosynthesis, including the enzymes in mevalonate pathway. Multiple JH biosynthetic-related genes have been identified from the genome or transcriptome data in insects, especially in the model insect species^36^. Whilst the genes encoding JH degrading enzymes and their functions have been fairly well characterized in agricultural pests^37^, this is not so for the JH biosynthesis steps.

Typically, the mevalonate pathway in most animals would lead to the production of steroids, and the regulatory mechanism of this pathway in vertebrates has been studied exhaustively^38^. In arthropods, the mevalonate pathway constitutes the initial step of JH biosynthesis, which has lost the ability to synthesize steroids^11^, and the peculiarities of mevalonate pathway in insects is the capacity to synthesize JH precursor. In this study, we successfully identify 10 mevalonate enzyme-encoding genes from a transcriptome of *H. armigera*. Previously, it has been reported that the mevalonate pathway is conserved among invertebrates and consisted of eight typical enzyme-catalyzed steps^11^. In our research, it is shown that each enzyme is encoded by a single gene, except for Fpps, which is comprised of four homologs. This pattern is similar to the study in *B. mori* ^14^ and *A. mellifera* ^39^, containing three and six Fpps homologs, respectively. However, Li *et al*. found two *Ace* isoforms and two *Hmgr* isoforms in *L. decemlineata* ^15^. Similarly, two transcripts of Hmgr were detected in crustaceans^40^ and two genes encoding Hmgs with 78% similarity have been reported in *Blattella germanica* ^41^.

Since the mevalonate pathway is generally considered as the first step of JH biosynthetic process, it was proposed that some enzymes in this pathway could be rate-limiting enzymes of JH biosynthesis^42^. The tissue expression profiles showed that 6 of the 11 mevalonate pathway genes were highly expressed in the head, similar to that of *JhAmt*. And the high expression of mevalonate enzymes in head can be attributed to the fact that CA is the special gland for the JH synthesis. Meanwhile, genes such as *HaMk, HaIppi, HaFpps*3*, and HaFpps*4 had varied expressions in tissues. And the non-specific tissue distribution of mevalonate enzyme-encoding genes was also found in *A. mellifera* ^39^, *D. punctate* ^43^, *A. aegypti* ^13^ and *B. mori* ^36^. This could be due to the fact that some defensive secretions and pheromones in insects are also included in the final products of the mevalonate pathway. In blister beetle *Epicauta chinensis*, the biosynthesis of a defensive toxin, cantharidin, plays a part in chemical defense as well as courtship and mating, is regulated by an enzyme in mevalonate pathway^18^. It is not surprising that some mevalonate enzyme-encoding genes have wide tissue expression in *H. armigera* as well as in other insects^44^.

A certain titer of JH is necessary for larvae to maintain the larval state, so in most cases the JH level increases during molting. High titers were found at the beginning of the 3^rd^, 4^th^ and 5^th^ larval instar in *B. mori*, and in the nymphal stage of *Cryptotermes secundus*, in which the JH titer rose before the next molt and then dropped sharply in both species^45,46^. In our study, JH levels were fluctuant during the developmental stages, and a transient increase of JH titer was observed right before the molt of each larval instar. The highest JH titer was detected on the 3^rd^ day of the 2^nd^-instar, while minimal amount of JH was detected in the pupal stage (Fig. 4A). Temporal expression patterns showed that 10 of 12 tested genes were significantly up-regulated in the late 2^nd^-instar larvae when JH level reached the peak. In the 5^th^ instar, the expression of *JhAmt* and most of the mevalonate enzyme-encoding genes declined to a low level, coupled with the decline in JH biosynthesis. This decline may be due to the shutdown of these genes. In *B. mori*, the levels of Mk, Pk, Dpp, and Ippi transcripts were correspondingly low when JH decreased during the pupal stage^14^. All these data show a tight correlation between JH titer and transcription regulation of the JH synthetic pathway genes.

Many factors regulate the mevalonate pathway and JH synthesis in insects, and some studies have shown that JH itself is a key regulator of JH pathway. In *L. decemlineata*, the activities of enzymes catalyzing the final step of JH biosynthetic pathway were diminished after JH III treatment^47^. In *Diploptera punctata*, the JH synthesis activity of the original CA was reduced when implanted with additional CA or ingested with exogenous JH *in vivo* ^48^. Methoprene, an analogue of JH, has been proved to activate JH pathway to regulate gene expression in *Drosophila melanogaster* ^49^ and *Nilaparvata lugens* ^50^. In our study, ingestion of methoprene significantly suppressed the expression of eight mevalonate enzyme-encoding genes as well as JhAmt. Similarly, the expression of Fpps was restrained by exogenous JH treatment in *Ips pini* ^44^, and *JhAmt* transcription was also repressed in *L. decemlineata* ^51^. However, in the last-instar larvae of female *B. germanica*, exogenous JH had no effect on *Hmgr* mRNA levels. In our results, no significant change in *Hmgr* levels was detected after JHA application, whereas JH increased the mRNA levels of *Hmgr* and *Hmgs* in *Ips paraconfusus* and *Dendroctonus jeffreyi* ^52,53^. Moreover, we found that ingestion of JHA significantly upregulated the transcription of *Jheh*, and the action of Jheh in JH degradation has been explored in certain insects^54–56^. Actually, the JH titer in insects is controlled by a balance of various processes including synthesis, degradation, sequestration and excretion. Transcription and activities of the biosynthetic and catabolic enzymes in JH pathway are affected when out of balance.

Four different isoforms of Fpps were identified in *H. armigera*, which was similar to that in Lepidoptera^36^ and Hymenopteran^39^, however, only a single-copy gene was identified in other insect orders^15,22^. Most insects produce only one chemical form of JH, but the Lepidoptera produce four derivatives that feature ethyl branches. In Lepidoptera, FPPSs have long been suspected of exhibiting structural features allowing them to accommodate the bulkier homologous substrates and products used as precursors of ethyl-branched JHs, therefore, it was speculated that the diversity of JH types is linked to the duplication of Fpps genes in Lepidoptera insects^57^. Phylogenetic analysis revealed that the Fpps family was conserved in insects (Fig. S2) with two aspartate-rich motifs: FARM and SARM. Interestingly, the FARM in most known eukaryotic Fpps had two aromatic residues which were not found in FARM of Fpps in many insects. A multispecies comparison of Fpps genes revealed that only several lepidopteran species contained the NDXXE signature motif^54^, which may have evolved a specialized function in the biosynthesis of JH.

Previously, functional analysis of JH biosynthetic genes was mainly focused on JhAmt and Hmgr, which have been shown to be essential in insect development and reproduction^15,16,35,43,58–60^, while few studies were about Fpps. In our study, the temporal expression of *HaFpps* was correlated with the changes of JH titer in larval stage due to the fact that their peak transcriptions were right before the arrival of the highest JH titer (Figs 3 and 4). Additionally, we found that knockdown of *HaFpps4* significantly downregulated the transcription of *HaAce*, *HaHmgs*, *HaMk*, *HaIppi* and *JhAmt* in *H. armigera*. One possible explanation was that RNAi treatment in *H. armigera* inhibited the production of isoprenoid, an essential JH precursor, resulting in a negative feedback and downregulating the expression of other genes to balance the quantities of intermediates and enzymes. In *A. aegypti*, FPPase efficiently hydrolyzed FPP into farnesol, and silencing of AaFPPase-1 caused a significant decrease in JH biosynthesis, which strengthened the possibility that FPP acted as the crucial enzyme in regulating JH synthesis^22^. Similarly, the activity of *A. aegypti* Mk exhibited efficient feedback inhibition from long-chain isoprenoids, such as geranyl diphosphate and farnesyl diphosphate^61^. This study also showed that knockdown of *Fpps*4 caused a significant decline in JH titer, leading to the darkening of cuticle and the molting disturbance in larvae (Fig. 6). These data suggest FPP might play a critical role in the synthesis of isoprenoids in insects, and the change of Fpps transcription can influence JH biosynthesis.

In conclusion, the mevalonate pathway is essential for the precursor synthesis of JH. Even though data on mevalonate pathway has been characterized in several insect species, there remained many unanswered questions in *H. armigera*. Here, our study annotated 10 genes in mevalonate pathway and investigated the function of particular genes in JH biosynthesis. This paper enriches the knowledge of regulatory mechanisms of JH biosynthesis in *H. armigera*. In addition, the functional analysis of HaFpps4 demonstrated that HaFpps4 played an indispensable role in JH biosynthesis, and was critical for regulating the transcription of JH pathway genes. Given the important role of JH in insect, genes involved in JH biosynthesis could provide potential targets for developing novel insecticide with high selectivity. And our results highlight the potential application of these genes for controlling this pest.

## Materials and Methods

### Insects

*H. armigera* used in this study were reared in the laboratory at 27 ± 2 °C, 75 ± 10% relative humidity with a photoperiod of 14: 10 (L: D). The larvae were reared on an artificial diet in the 24-well plate, and transferred into 25-ml glass tubes containing an artificial diet at the fifth instar for pupation (one larvae per tube). Adults were fed with 10% sugar solution.

### Tissue collection, total RNA isolation, and cDNA synthesis

For developmental expression analysis, individuals at the stages of egg, larvae (first, second, third, fourth and fifth instar), white pupae, green pupae and black pupae were collected, and samples from female and male after emergence were also collected. For tissue-specific expression analysis, tissues (including head, epidermis, fat body, midgut, malpighian tubules, hemolymph, and peritrophic matrix) were dissected from the 5^th^-instar 0 d larvae in phosphate-buffered saline (PBS). All samples were frozen immediately in liquid nitrogen and stored at −80 °C until RNA isolation. Each group contained 3 to 50 individuals and three biological replicates were performed.

Total RNA was extracted using Trizol reagent (Invitrogen, Carlsbad, CA). The quality of each RNA sample was measured by a NanoVue spectrophotometer (GE-Healthcare, Germany) and 1% agarose gel electrophoresis. After the digestion of residual genomic DNA with DNase I (Promega), 2 μg total RNA was reverse transcribed to cDNA with the Fast Quant RT kit (TIANGEN, Beijing, China) according to the instructions.

### Confirmation of genes involved in the JH biosynthetic pathway

Specific primers for gene cloning (Table 1) were designed based on homologous sequences identified in our transcriptome database. The sequence information was validated using cDNA of the whole body as templates for PCR. All PCR products were analyzed by 1% agarose gel electrophoresis, ligated with the pMD18-T vector (Takara, Dalian, China), and then transformed into competent *Escherichia coli* cells. After transformation, positive clones were picked for sequencing (BGI Tech).

**Table 1:** Primers used in this experiment.

Primer	Forward primer (5′-3′)	Reverse primer (5′-3′)	Size
**RT-PCR analysis**			
Actin	CCGTCCACAATGAAGATCAA	ATCGACAATGTTCCGCATTC	330
Ace	TGGCAACTGTGTTCCAAAAG	GAGAGCATGGCACAAGTGAA	407
Hmgr	GAGCACCGGCTATTAAGCTG	CAGAGTTTTGGCATCGGTTT	416
Hmgs	ATGGCAGAAAGGCGATAGTG	TCTGGTGATGCATTGAGGAA	406
Mk	TTACGGGCAAATTCTCTTGG	TGATGGAGATCCGTGCATTA	419
Pk	GTTGGGTGACGGAGAGTACA	TCCACCTCCTGTTTTCCTTCA	351
Ippi	CGGTGAGAGTGGAAAAGAGG	CAACTCTGGGTCCATCTGGT	413
Dpp	TGACTCTACACACTGGCCTG	TTGGTCCGGCATCAAATGTG	365
Fpps1	GTCTGATGGAAGTCCTGCAAAT	GGTCTCTGCTGCCGTAGTATTC	474
Fpps2	CCAGAGAACATAACGGAGGAGA	AGAGATATGGGCAGCTTGTACG	416
Fpps3	AATGGGACAACACTTGGACTTC	GGTAGCCTCAAATCACCGTAAA	426
Fpps4	CGAAGCCATCACGAAGTACA	CCTCTTGTGACTTGCTGGAT	426
**qRT-PCR analysis**			
Actin	TGGTATTGCTGACCGTATGC	CTGTTGGAAGGTGGAGAGG	142
Ace	TCCAAAAGGAGAATGGAACG	TCCCCATCAGCATATCCAAT	145
Hmgr	ATCGTGGCCACATTAGCTCT	CATCAGCGGCTTGGTACTCT	133
Hmgs	AAGGCAGAGTGTGGAGCCTA	GCCGGGGAACAGTATGTCTA	112
Mk	ATGCCCTGCAGAATAACCAG	CAGCTCCAATAGTTAACTCA	132
Pk	TCTGGAGTTGACGATGTTGC	AACCAGACAGCAATCCCAAT	133
Ippi	CGCTGAAACCACAGACTGAA	TTCCCACGAAGTTGTCCTTC	108
Dpp	TGTATACGGTGGCTTCGTCA	GCATTTCAGGCCAGTGTGTA	101
Fpps1	CTGCACACAGCACGATTTCT	TAGTCGCCGAAGTACCGACT	145
Fpps2	AAGGATTGCCAATGTTACGG	ATGGAACTCCACCCTTTCCT	109
Fpps3	CAGGAAGGTGCTTGAACACA	CTGGCCAGCTTGAGTTTCTC	123
Fpps4	GAGACTGGCAAGCACATTGA	CAGCATTTTGTAAGCCAGCA	123
**RNAi analysis**			
Ace	TAATACGACTCACTATAGGGTGGCAACTGTGTTCCAAAAG	TAATACGACTCACTATAGGGAGAGCATGGCACAAGTGAA	406
Fpps4	TAATACGACTCACTATAGGGACATCGTAGAAGGCACAGAGA	TAATACGACTCACTATAGGGTCTGTAAGGCGACAACTG	519
GFP	TAATACGACTCACTATAGGGGCAACATACGGAAAACTTACC	TAATACGACTCACTATAGGGTGTGTGGACAGGTAATGGTTG	

To analyze the genomic structure of identified genes, primer pairs were used to amplify the genome DNA of *H. armigera* (Table S2). Genomic DNA was extracted using TIANamp Genomic DNA kit (TianGen, Beijing, China) following the manufacturer’s instructions. PCRs were conducted using the LA Taq polymerase (TaKaRa, Dalian, China). The PCR products were subcloned into the pEasy-T3 vector (TransGen, Beijing, China) and sequenced.

### Quantitative real-time PCR

The expression profiles charactering different developmental stages and tissues were analyzed by quantitative real time PCR (qPCR). The cDNA templates were prepared as described, and primers for qPCR were designed by Beacon Designer 7.9 (Table 1). The reference gene *β*-*actin* was employed to calibrate the sample-to-sample variation and normalize the target gene expression. The amplification efficiencies of the tested genes and reference gene (*β-actin*) were calculated using a gradient dilution of templates. Results showed that the amplification efficiencies for tested genes and reference gene were similar and close to 100%. The qPCR reaction was performed using the SYBR® Premix Ex Taq^TM^ II kit (Takara, Dalian, China) and PCR procedure was as follows: 95 °C for 30 s, followed by 40 cycles of 95 °C for 5 s, and 60 °C for 20 s, and melting curve stage. Negative controls without template were included in each experiment. To check the reproducibility, a total of three biological replicates were analyzed and each biological replicate was assessed three times. Relative expression levels were calculated by the following formula: R = 2^−(ΔCt sample-ΔCt calibrator)^, where R is the relative expression level, ▵Ct sample is the average difference between the Ct of the gene and the ß-actin in the expreimental sample, and ▵Ct calibrator is the average difference between the Ct of the gene and the ß-actin in the calibrator. A representative sample was set as the calibrator. All methods and data followed guideline of the MIQE (Minimum Information for publication of Quantitative real time PCR Experiments)^62^.

### Quantitative determination of JH

To determine the JH levels, individuals at the different stages were sampled just as that for qPCR analysis. For JH extraction and JH determination, the methods were operated as described in previous paper^63^. Briefly, the samples were weighed and frozen immediately in liquid nitrogen. Then the samples were homogenized by grinding in 0.9 ml of methanol/isooctane (1:1, v/v), and subsequently adding 1 ml of n-hexane. The resulting suspension was treated with ultrasound (100KHZ) for 5 min and further centrifuged (10 min, 12,000 × g at 4 °C). Next, the hexane (upper) phase was removed by pipette. Another 1 ml of n-hexane was added to the tube and treated ultrasonically before centrifugation and the removal of the hexane (upper) phase. This process was repeated three times. The hexane phase was then ready to dry completely under Termovap Nitrogen Sample Concentrator (HP-5016SY). Finally, 500 µl of MeOH was added as a solvent, and the samples were stored at −80 °C. Six parallel samples were prepared as the biological replicates.

At first, a five-point calibration curve of JH III (Sigma, USA) was used as the standard (Figs S6–S7). Level of JH III was measured using high performance liquid chromatography (HPLC) (1200 series, Agilent Technologies, San Jose, CA) and the system was operated using LC 3D B.04 software. Chromatographic separation was carried out at 30 °C in the isocratic mode using methanol (waters) (80:20, v/v) as the mobile phase. The injection volume was 20 µl in partial loop with needle overfill. The column used was a reverse phase C18 chromatographic column (ZORBAX SB-C18, 250 nm × 4.6 nm, Agilent Technologies) at a flow rate of 800 µl/min. A total separation of 30 min was needed. Each measurement is performed three times.

### Effects of methoprene application on genes transcription

The JH analogue methoprene was used to analyze whether JH could affect the transcription of genes in JH synthetic pathway. Methoprene was selected because of its ability to trigger many of the same responses as JH, and it was more stable in hemolymph therefore prolonging the physiological effects. Methoprene stock was diluted to 5 mg/ml with DMSO, and 1 ul methoprene solution (2 mg/ml) was injected into the newly molted 4^th^ instar larvae (2 µg/larva). In the same way, an equivalent volume of DMSO was injected into the control group larvae. Each group contained at least 30 individuals with three biological replicates. To determine the effect of methoprene on transcription response in the JH biosynthesis pathway, total RNA from the head tissues in both the hormone treatment and control group was extracted at 24 h after injection, and qPCR assay was performed as before.

### Synthesis of dsRNA and RNA interference

Synthesis and microinjections of dsRNA were performed as previously described^64^. In short, target fragments of HaFpps4, HaAce and GFP (green fluorescent protein) for dsRNA synthesis were amplified using specific primers. The PCR products were sub-cloned into the pGEM-T vector and used as templates for target sequence amplification. The dsRNAs were synthesized *in vitro* using a HiScribeTM T7 Transcription Kit system (New England BioLabs, Ipswich, MA) with specific primers fusing the T7 promoter at the 5′ end and dissolved in DEPC water. The purity and yield of the dsRNA was checked on a 1.0% agarose gel and a spectrophotometer, respectively. The primers used for dsRNA synthesis are presented in Table 1.

In each knockdown experiment, 60 individuals of newly molted 4^th^-instar larvae were cold-anesthetized, and 5 μg single dsRNA solution (ds*HaFpps4*, ds*HaAce* or dsGFP) was injected into the abdomen of each larva using a microsyringe (Hamilton, Bonaduz, Switzerland). Each treatment was performed with three biological replicates. Subsequently, ten individuals were randomly selected at 24 h, 48 h, 72 h and 96 h after injection, then the tissues of head, epidermis and midgut were dissected. To investigate the RNAi efficiency, qPCR analysis was performed as mentioned, and the levels of JH were measured to assess the effect of RNAi on JH biosynthesis.

### Expression of HaFpps4 genes in insect cells

The *Trichoplusia ni* BTI-Tn-5B1-4 (Hi5) cell line was employed to investigate the subcellular localization of HaFpps4. Cells were incubated in Grace’s insect cell culture medium (Life Technologies Co., Grand Island, NY) supplemented with 10% fetal bovine serum (Life Technologies Co., Australia), 100 unit/ml penicillin, and 100 mg/ml streptomycin (Life Technologies Co., Grand Island, NY) at 28 °C^65^.

The whole open reading frame of *HaFpps4* gene was amplified and ligated with the pGEM-T vectors. The target fragment was excised with restriction enzymes (Table 1) from the recombinant plasmid, and then subcloned into the plasmid p-EGFP-N1 at the corresponding sites to generate plasmids pHaFpps4-GFP. The Hi5 cells were dispensed into 6-well plates (Corning, USA) at the density of 1 × 10^6^ cells/well and grown overnight. The recombinant plasmid of pHaFpps4-GFP was transfected into Hi5 cells at 2 mg/well using cell-fectin reagent (Life Technologies), as previously reported. At 24 h after transfection, cells expressing recombinant proteins were washed with phosphate-buffered saline, fixed in 4% paraformaldehyde for 20 min, and stained with Hoechst 33342 (1 μg/ml) for 20 min at room temperature. Then, cells were photographed using an inverted fluorescence Nikon microscope (TE2000-S, Japan).

### Data analysis

All the data in this study are presented as means ±SE. Significant differences were determined by one tailed student t-test or one-way analysis of variance (ANOVA) followed by a least significant difference test (LSD) for mean comparison. All statistical analysis was performed with SAS 9.20 software (SAS Institute, Cary, NC).

## Electronic supplementary material

Supporting information
