# Supplementary material for: Identification and characterization of genes involving the early step of Juvenile Hormone pathway in Helicoverpa armigera
Source: Sci Rep. 2017 Nov 29;7:16542. doi: 10.1038/s41598-017-16319-z (PMC5707400; doi:10.1038/s41598-017-16319-z)
Supplement: Supplementary file 1 — Supporting information [file 41598_2017_16319_MOESM1_ESM.pdf]

Supplementary Information for

Identification and characterization of genes involving the early step of Juvenile Hormone pathway in *Helicoverpa armigera*

Wanna Zhang1,2*, Long Ma3*, Haijun Xiao1, Chen Liu2, Lin Chen2, Shaolong Wu4，Gemei Liang2

1 Institute of Entomology, Jiangxi Agricultural University, Nanchang, 330045

2 State Key Laboratory for Biology of Plant Diseases and Insect Pests, Institute of Plant Protection, Chinese Academy of Agricultural Sciences, Beijing 100193, China

3 Jiangxi Key Laboratory of Bioprocess Engineering and Co-Innovation Center for In-vitro Diagnostic Reagents and Devices, College of Life Sciences, Jiangxi Science & Technology Normal University, Nanchang 330013, China

4 China Tobacco Midsouth Agricultural Experimental Station, Changsha 410128, China

* These authors contributed equally to this work.

Correspondence and requests for materials should be addressed to G.-M. L. (gmliang@ippcaas.cn)

Supplementary Figures

Supplementary Figure 1： Diagram showing the JH biosynthetic pathway (modified from Bellés et al[11] and Noriega et al[10]). The mevalonate pathway is presented in the dashed box. Precursors are in bold and connected by arrows. Enzymes are in italics.

Supplementary Figure 2： Phylogenetic analysis of prenyltransferases from selected insects. The protein names and accession numbers used in this analysis are as follows: AgFpps: *Anopheles gambiae* (EAA04004); DaFpps: *Dendroctonus armandi* (ALF44684.1); TcFpps: *Tribolium castaneum* (NP_001164089.1); EgFpps: *Epicauta gorhami* (AHH34857.1); DmFpps: *Drosophila melanogaster* (NP_477380); DbFpps: *Drosophila busckii* (XP_017837938.1); HlFpps: *Habropoda laboriosa* (KOC62899.1); MpFpps1: *Myzus persicae* (ACA48701.1); MpFpps2: *Myzus persicae* (ABY19313.1); BmFpps1: *Bombyx mori* (BAF62113.1); BmFpps2: *Bombyx mori* (NP_001093301.1); BmFpps3: *Bombyx mori* (NP_001093302.1); MuFpps: *Mythimna unipuncta* (AAY33487.1); ObFpps: *Operophtera brumata* (KOB70829.1); CfFpps: *Choristoneura fumiferana* (AAY33486); PmFpps: *Papilio machaon* (KPJ18340.1); PxFpps: *Papilio xuthus* (KPJ05696.1).

Supplementary Figure 3： The expression levels among *HaFpps* genes in different tissues. He: heads; Ep: epidermis; Fb: fat body; Mg: midgut; Mt: malpighian tubules; Pm: peritrophic matrix. The bars represent the average (±SE) of biological repeats.

Supplementary Figure 4： Subcellular localization of HaFpps4-GFP in insect cells. The images were taken at 24 h post-transfection with the plasmid pHaFpps4-GFP. I, II and III represented three detected types.

Supplementary Figure 5： The full-length gels for each gene presented in Figure 2.

Supplementary Figure 6: The specificity of the qPCR primers

Supplementary Figure 7： Standard curve of JH-III pure chemical

Supplementary Figure 8： The chromatograph of JH-Ⅲ pure chemical (100 ng/μl) and sample (A standard, B sample)

Supplementary Tables

Supplementary Table 1： Enzyme-encoding genes in JH biosynthetic pathway in *H. armigera*

Supplementary Table 2： Primers used for the genomic sequence analysis.

Supplementary sequences

Data set 1: Sequences of genes identified in our study

Supplementary Figure 1

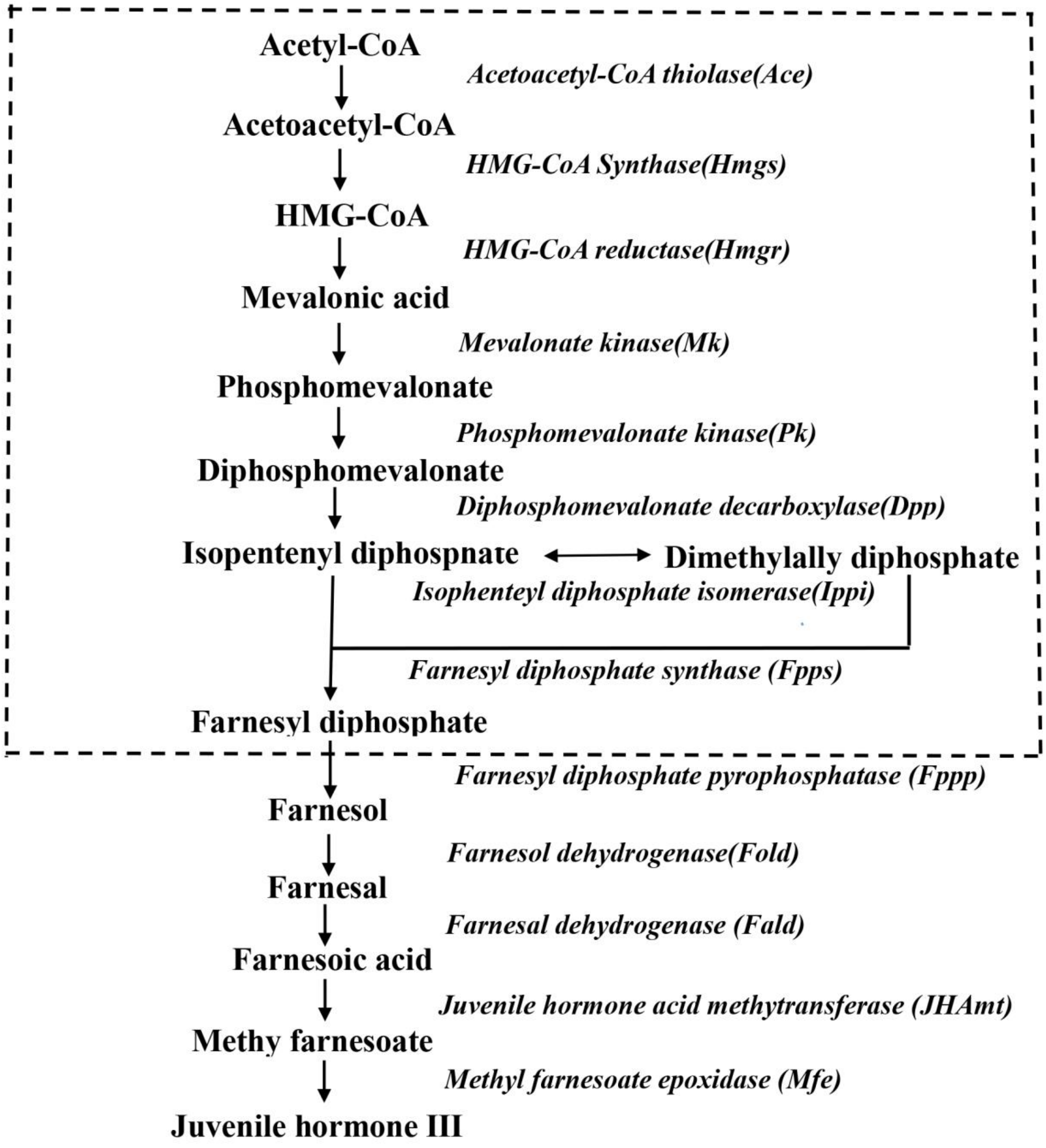

Supplementary Figure 2

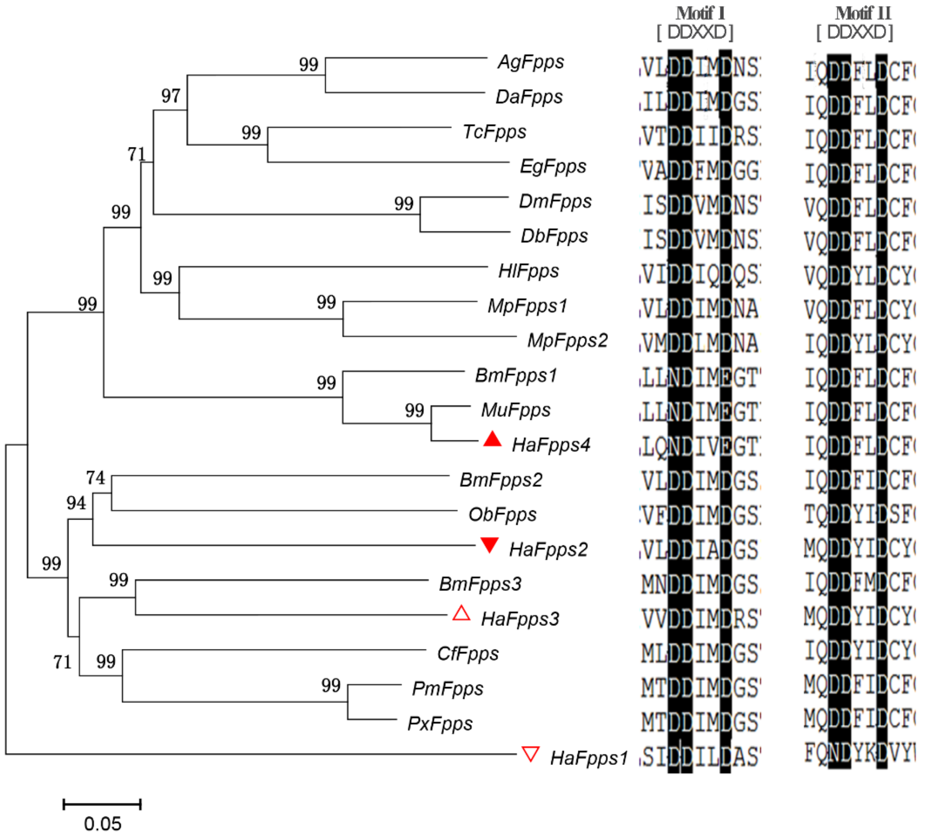

Supplementary Figure 3

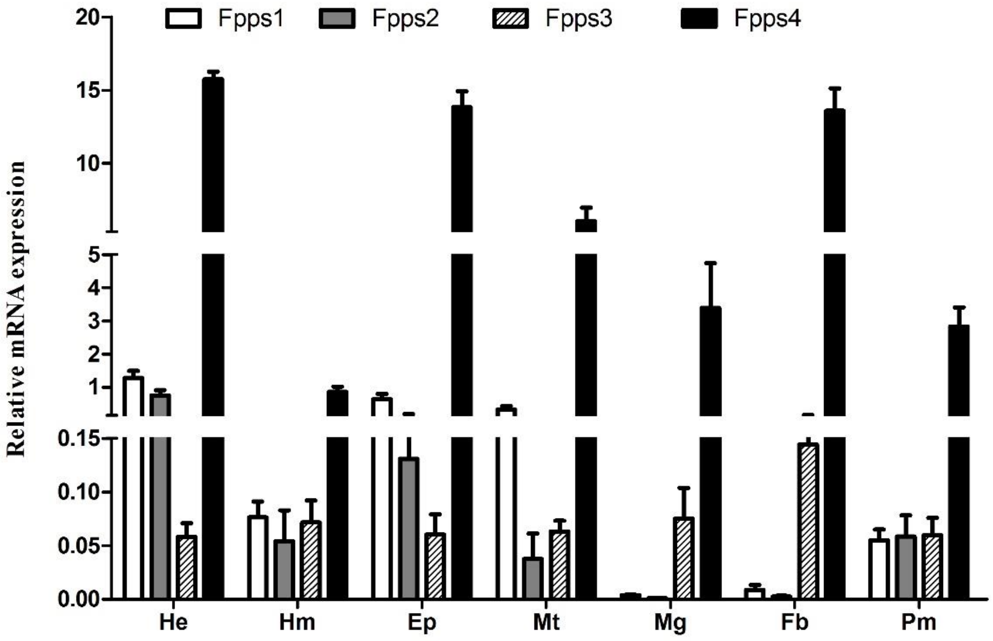

Supplementary Figure 4

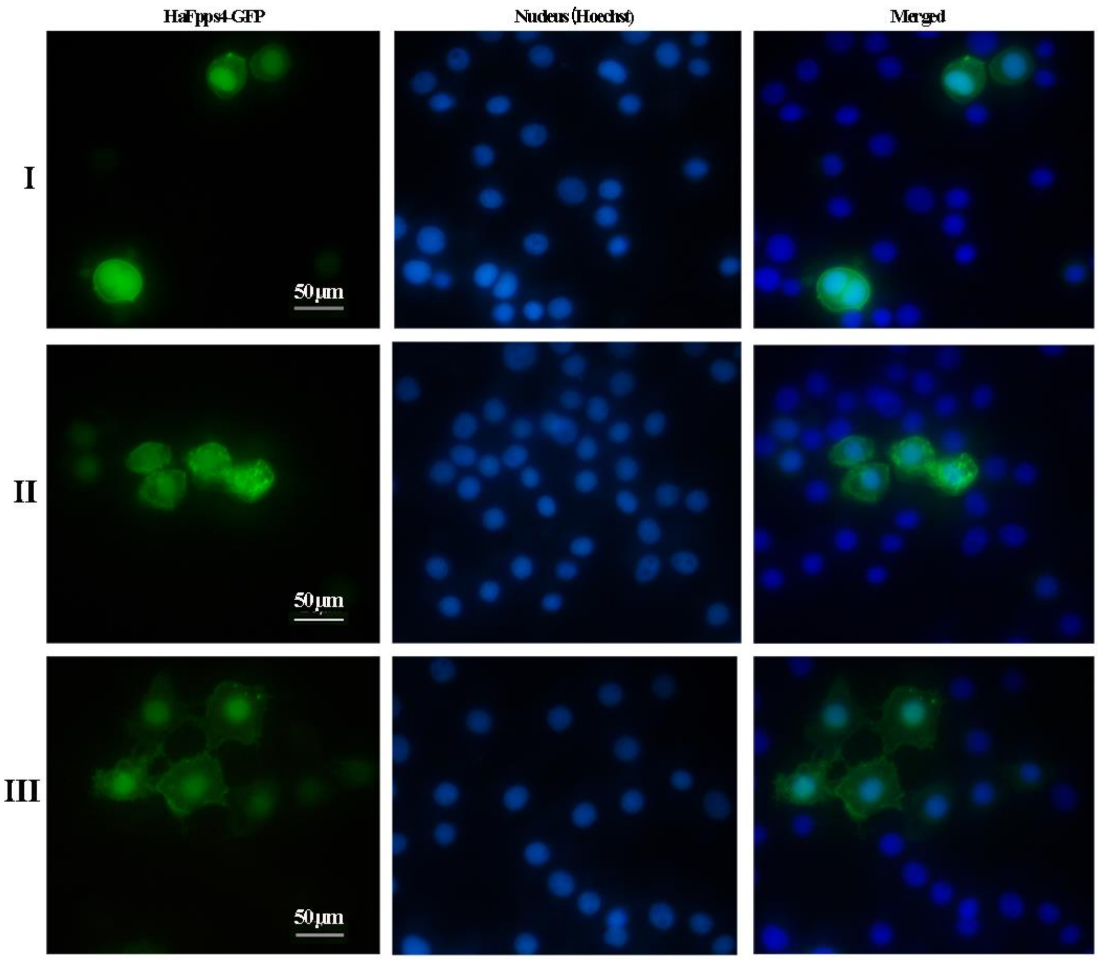

Supplementary Figure 5

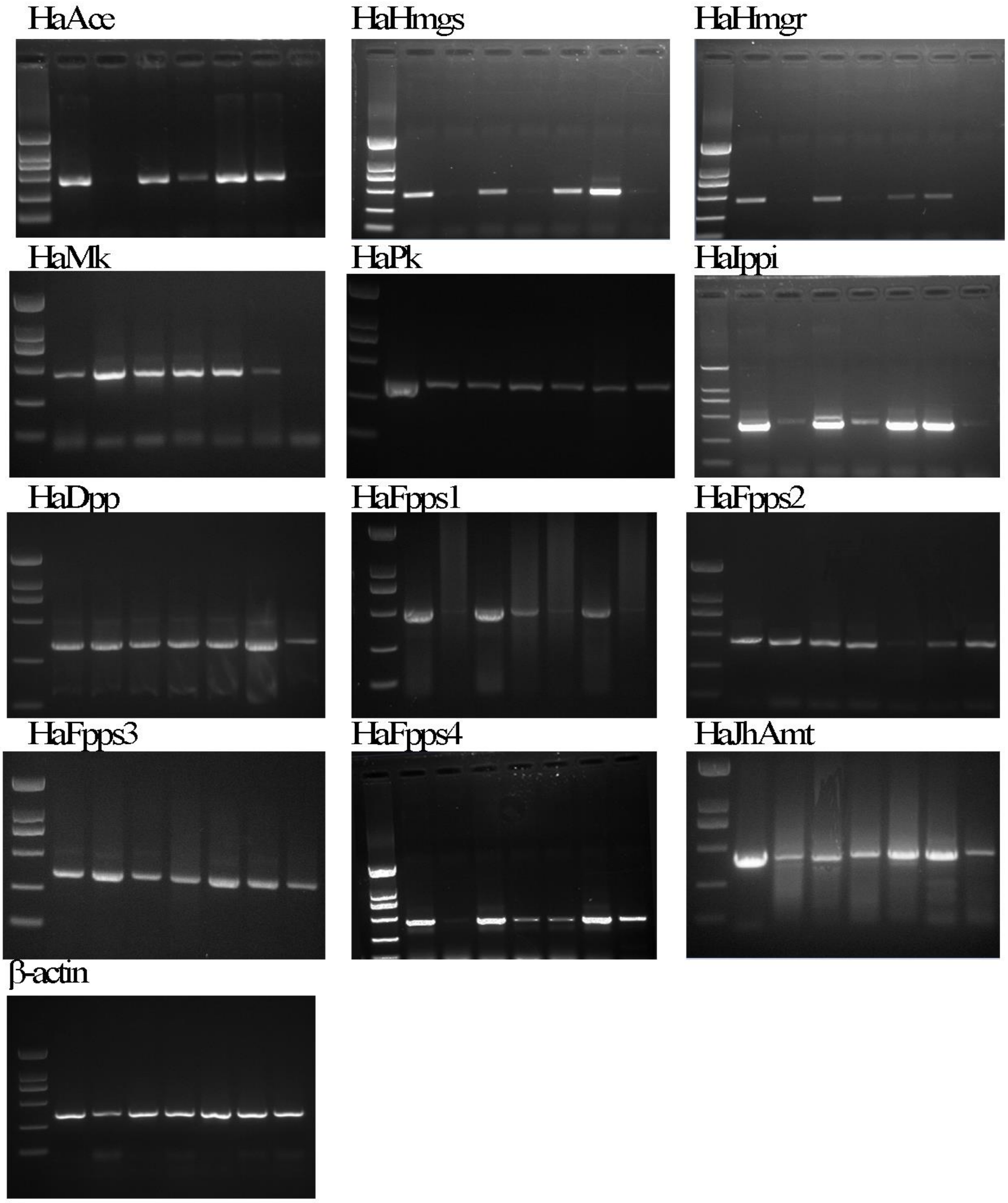

Supplementary Figure 6

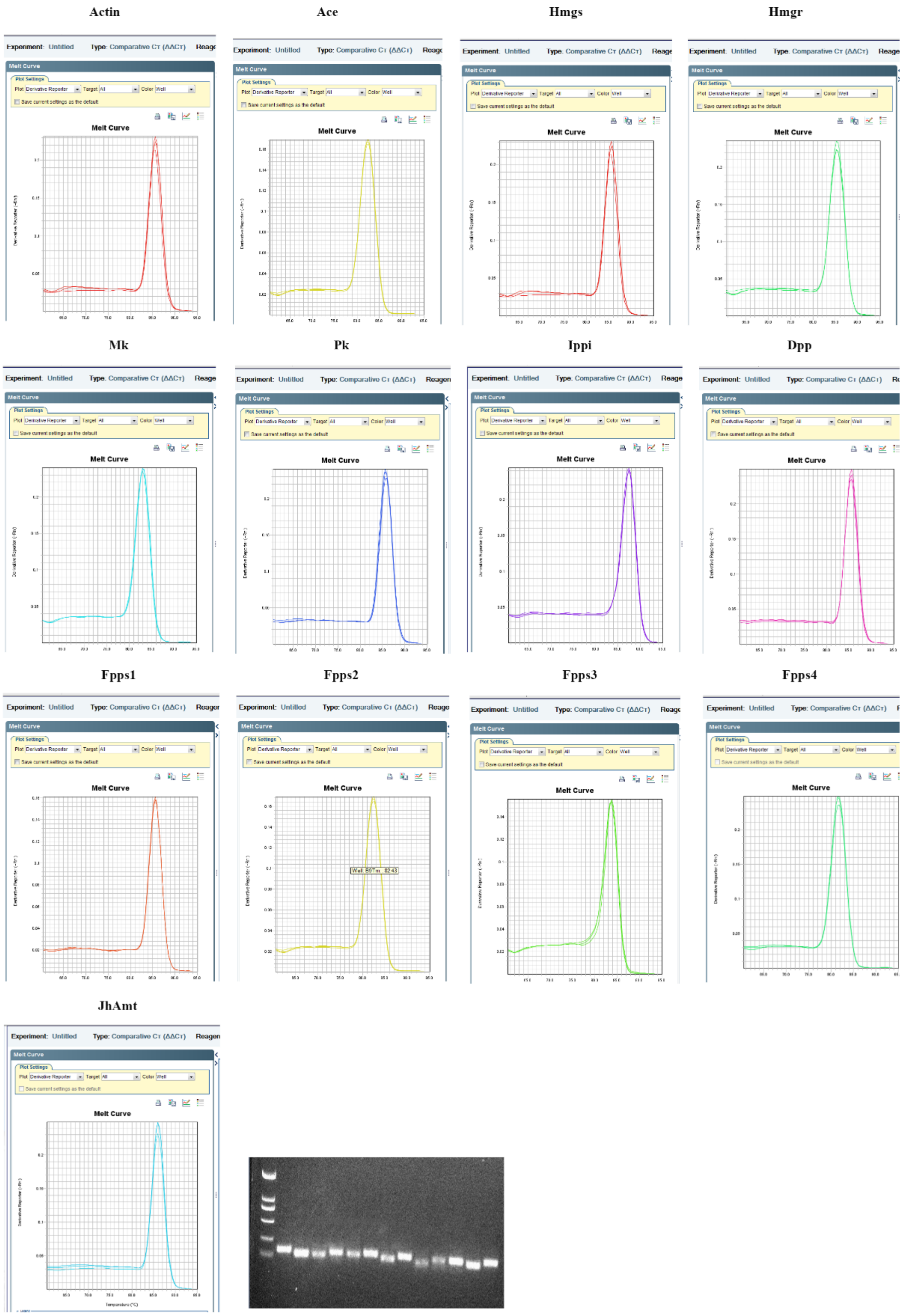

Supplementary Figure 7

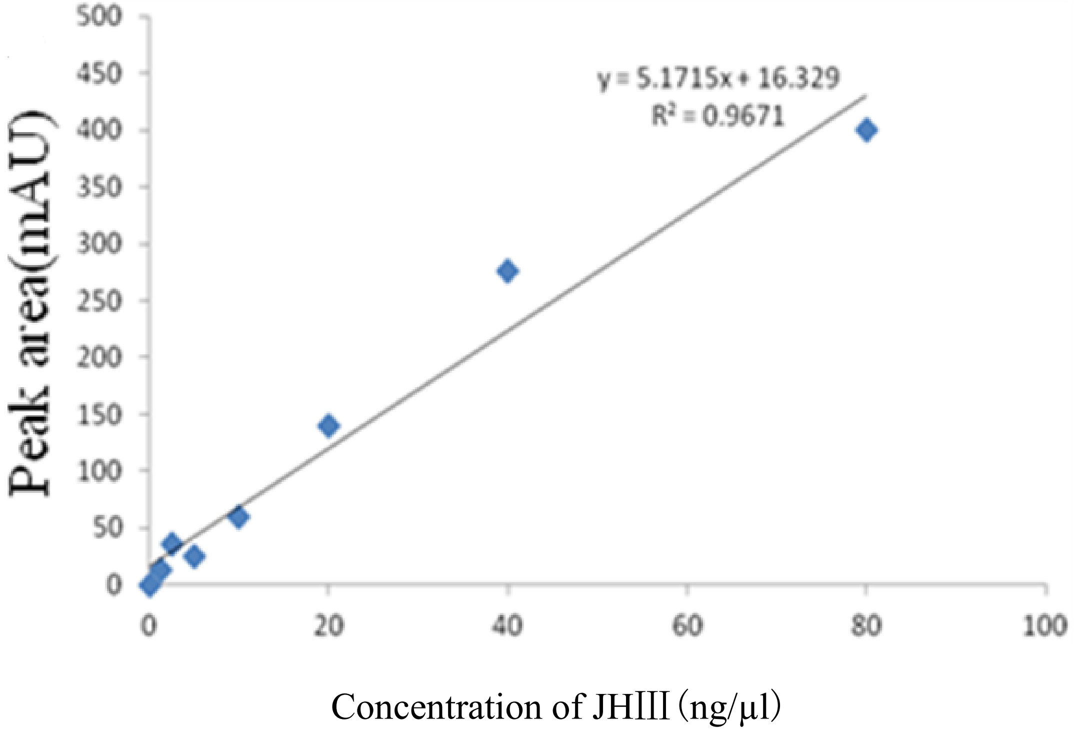

Supplementary Figure 8

A

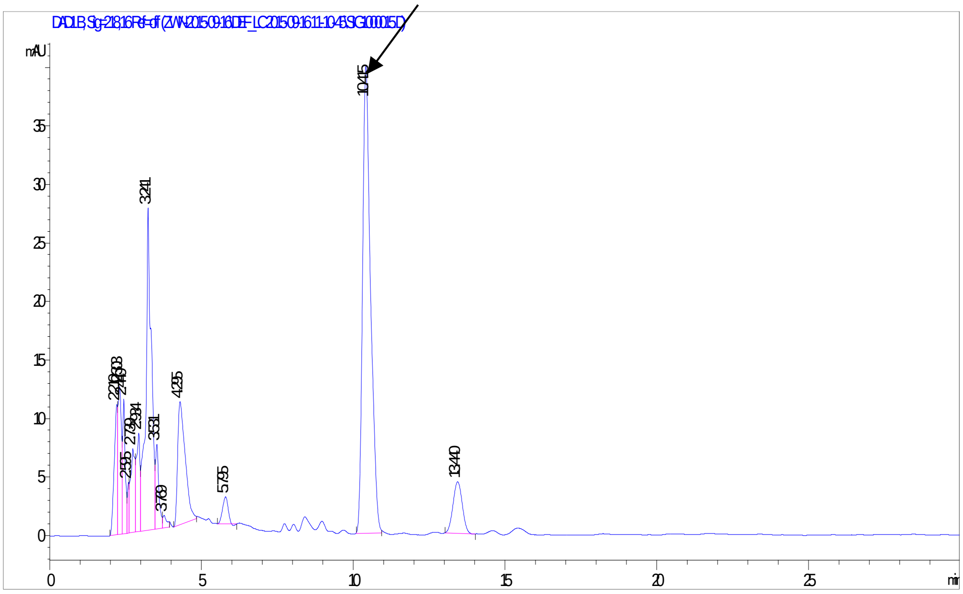

B

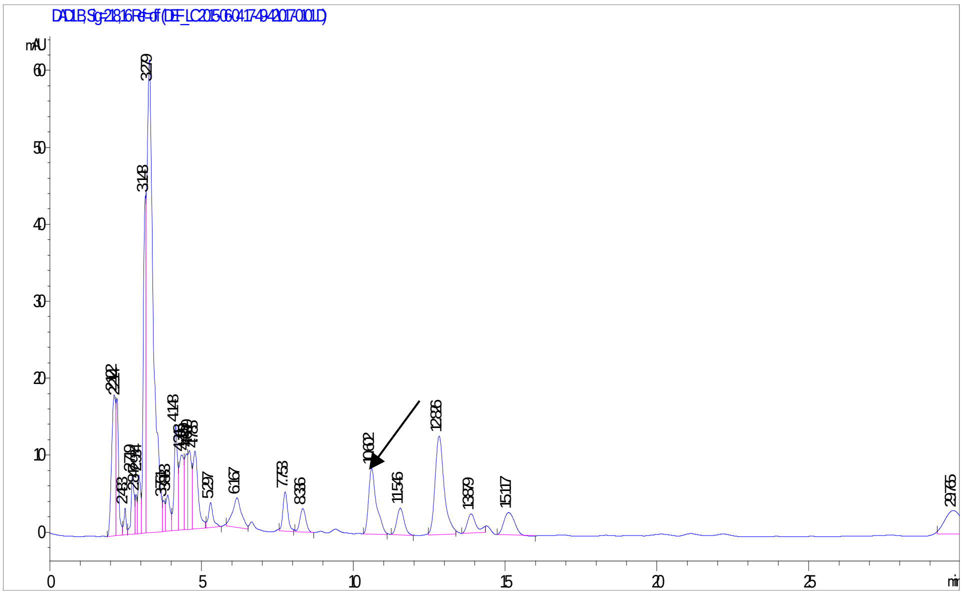

Supplementary Table 1

Gene	Accession number	ORF(bp)	Peptide(aa)	Function	Identity	E-avlue
HaAce	KY71766	1272	423	catalyze 2 molecules of Acetyl-CoA to form acetoacetyl-CoA	81%	0.0
Hmgs	KY71767	1371	456	catalyze Acetoacetyl CoA+acetyl-CoA to form 3-hydroxy-3-methylglutaryl CoA, HMG-CoA	87%	0.0
HaMk	KY71768	1242	413	Phosphorylates mevalonate	83%	0.0
HaPk	KY71769	558	185	Phosphorylates phosphomevalonate	75%	$2e^{-87}$
HaDpp	KJ951986	1161	386	catalyze decarboxylation of diphosphomevalonate to form Isoamyl enol pyrophosphate	75%	0.0
HaIppi	KJ951988.1	759	252	isomerization of IPP to DMAPP	86%	$4e^{-166}$
HaFpps1	KY71770	1131	376	sequential condensation of IPP with DMAPP and then GPP to form FPP	39%	$3e^{-87}$
HaFpps2	KY71771	1164	387		51%	$1e^{-135}$
HaFpps3	KY71772	1155	384		57%	$3e^{-165}$
HaFpps4	KY71773	1284	427		94%	0.0

Supplementary Table 2

Name of Primers	Forward Sequences（5'-3'）	Reverse Sequences（5'-3'）
Ace-1	TTTTTTTTGAAAGGCAATAGA	TATTCTCAGCACAGTTACCCAT
Ace-2	TTTTTTTTGAAAGGCAATAGA	CCACCATTACAGACGGCA
Hmgr	ATGAAAGTCTGGGGAGCT	TAAATGTCTGCTGAGCTGCAAAC
Hmgs	ATGGGCGGAAGAGTTGA	TCAAACTTTCCTTGCATAA
Ippi-1	TCATTCTTTGTTGGGCTTTAC	GCAGCACTTCACCATCATCTC
Ippi-2	CAACTTCGTGGGAACTGCTA	TGAGACGATGAAGGTTGTCCC
Dpp	TCAAAGTAGTTTAGTAAATTATCGCTG	TTCTAAGAATATAAGATTTCAT
Mk-1	CGCAAAAGACTCTCACGGTT	TAATGATGTGTAGGAACTCGTCG
Mk-2	GTGGAGCCAACGATACGGAT	ATGCTGCCACCTTCACGA
Mk-3	TCGCTCATACAGCCACGC	TCAATCTCACACTTGCCACTG
Mk-4	TGAGATTGATAGTGAAGCGGA	CCGAGAGCAGACAGTGGTT
Mk-5	CCACTGTCTGCTCTCGG	GCTCACGATTCACTCACCCAT
Pk	TTGCTATTCAGCGGTAAGAGA	ATCATAAACCAGACAGCAACC
Fpps1-1	ATGGCCCTTTTGCTCCGTAA	GCAGTCATTAGTCGCCGAAGTA
Fpps1-2	TACTTCGGCGACTAATGACTGC	TTAATAATCTCTCAGTCGGCACG
Fpps2-1	TGCTGTTGGGAAAAGGATTAC	CACTATAAAGTCTCTGAAAGGT
Fpps2-2	ACCTTTCAGAGACTTTATAGTG	TCATGTTCTTACCGCCGATTA
Fpps2-3	TAATCGGCGGTAAGAACATGA	CTTTATTCGTTCTATGTGTGCC
Fpps3-1	AGAAAGTGAGCACGTAGCTAATATT	CTGGCAGAGCATCGTGAAA
Fpps3-2	AACTCCCTGAAGTGGGCAAT	AAGTCCAAGTGTTGCCCCAT
Fpps3-3	ATGGGGCAACACTTGGACTT	GTGACTGATTCGTCGCCGTA
Fpps3-4	ATGGGGCAACACTTGGACTT	GTAGGAGCGGTTTCAGAGGG
Fpps4-1	GCGAACAGACGCTACACCTA	GTAGCCATCGGCATTTCCT
Fpps4-2	ACGCAGGAAATGCCGATG	TGTAAGCCAGCACTGTCGC
Fpps4-3	ACGCAGGAAATGCCGATG	AGACCGACTTCTGGGTGCTT
Fpps4-4	CACAGTTCCACACTCACCAG	GAACAGGGTAAGGTCGGGCT
Fpps4-5	CACAGTTCCACACTCACCAG	CTGTAAGGCGACGACTGCGA

Data Set 1

>Helicoverpa armigera Acetyl-CoA thiolase

ATGTTTTTTTTGAAAGGCAATAGAATTATTACCATCAAAATGCGGCCAGCATATAATAAGCTTTATAAAG
CGATGGCTGCGTATTCTTCAAAACTTTCCCTGAACGATGTGGTGATCGCGTCCGCTGTGAGGACTCCA
ATTGGCTCCTTTAAGGGTAGCCTGTCGAGCCTTTCAGCTACCGAACTTGGAGGTGTCGCCGTTAAAGC
AGCAGTTGAAAGAGCCGGCATTCCTGAGGAAGAAGTTAAAGAGGTATACATGGGAAATGTCTGTTCT
GCTGCTTTAGACCAGGCTCCTGCGAGGCAAGCTACCATTTTCGGAGGTCTTCCTAAAAGCACAATCTG
CACAACTGTTAATAAAGTTTGTTCATCTGGCATGAAGGCCATCGTATTGGCCACGCAAGGTTTGCAAA
CTGGAACTCAAGATGTGATATTAGCTGGAGGAATGGAATCTATGTCAAACGTGCCTTACTATTTGAAGA
GGGGTGATACACCATATGGAGGTGTTCAGTTGAATGATGGCATTTTGTATAATGGCTTGACTGATGTTT
ATAACAAAATCCATATGGGTAACTGTGCTGAGAATACAGCAAAGAAACTGAACATCTCAAGGAAGGA
ACAAGACGACTATGCTGTTTCAAGTTACAAGAGAAGCGCTACAGCATATGAAAATAAGACTTTTGCTG
ATGAGCTGGTACCAGTACCGGTGCCCCAGAAGAGAGGTGCTGCCCCAGTTATCTTTGCAGAAGATGA
GGAGTACAAGAAGATCAACTTTGATAAATTCACCTCATTGGCAACTGTGTTCCAAAAGGAGAATGGA
ACGGTGACAGCTGGTAATGCCTCAACATTGAATGATGGTGCTGCAGCCATGGTCCTGATGACTGCTGA
TGCTGCTCAAAGATTGAATGTTAAGCCCCTGGCTCGTGTTATTGGATATGCTGATGGGGAATGTGACCC
TATTGACTTCCCAATTGCTCCAGCAGTTGCTATTCCAAAATTGCTAGCTAAGACTGGTGTCAAGAAAG
AGGATGTAGCTATGTGGGAAATTAATGAAGCCTTCAGTGTTGTAGCTTTGTCCAACATGAAAATGCTA
GAACTTGACTCTAACAAGATTAACATTCATGGAGGAGCTGTTAGTCTTGGCCATCCCATTGGTATGTCT
GGTGCCAGAATTGTTGTTCACTTGTGCCATGCTCTCAAGAAGGGCGAAAAGGGTGTAGCTGCTATCTG
TAACGGTGGAGGTGGTGCCACATCTATCATGATTGAGAAATTGTAA

>Helicoverpa armigera Hydroxymethylglutaryl-coenzyme A synthase

ATGGGCGGAAGAGTTGAAAACGTTGGTATACTCGCCATGGAGTTATACTTCCCTTCACAATACGTAGA
CCAAACGGAACTGGAAAAATTTGACGGCGCTTCCACCGGCAAGTACACAGTCGGCCTCGGGCAGCT
CAAAATGGGCTTTTGCTCTGACAGGGAAGACATAAACTCTATTTGTATGACAGCTCTGCACAGACTGA
TCGAGAAAAACAGCATAAATCTTCATGACATTGGCCGGCTAGAGGTTGGTACTGAGACAATCATTGAT
AAAAGTAAGAGTGTGAAGACCTACCTCATGACCTTGTTTGCTGAGGAAGGTGCAACTGATATTGAAG
GTATTGACACAACCAATGCTTGCTATGGAGGTACTGCAGCTCTGTTTAATGCTGTGAACTGGGTGGAG
TCATCTTCCTGGGATGGCAGAAAGGCGATAGTGGTGGCAGGAGACATTGCAGTGTACGGCAAGGGGC
CAGCGAGGCCCACAGGTGGGGCAGGAGCTGTGGCCGTGCTCATAGGGCCCAATGCTCCCCTGGTACT
TGACTGTGGTGTCCGTGCTTCATACATGACTCATGCTTATGACTTCTACAAGCCAGACTTGGCATCAGA
GTTCCCATTTGTTGATGGGAAGCTCTCCATTAAATGCTACCTCAGTGCTTTAGATAATTGTTACAATTTG
TATTGTAAGAAGATGAGGAAGATAGATCCTGACTTCAAAGGCTTATTGAGCTTAGATGGTATGTTGTTC
CACTGTCCATACAGTAAACTAGTACAGAAGTCCCTAGCTAGAGTGTGTTTCAACGATTTCCTCAATGC
ATCACCAGAGCAAAGAGAAAAACAGTTTCCAGGTTTATCCAACTTCAGTTCACACAAATTGGAGGAA
ACATACTTTGATAGAGATGTAGAAAAAGCTTTCATGACATACAGCGAGGCTCTGTTTGAAGAGAAGAC
CAAGCCATCACTGTATATAGCAAAGAATGTAGGAAACATGTACACACCATCACTGTATGGAGGCCTTG
CTTCATATCTCATTAGTAAATCTCCTGAGCAGCTGATTGGGAAGAAGTTTGCACTATTTTCATATGGTTC
AGGTCTGGCCTCAACTATGTATTCAATAAATATTTGCAATGACATGAGTGTTGGTTCAAAGTTGGCAAA
ACTGTTGAGTTCATTGAATGAAACAGTTTCTTTATTAAATGAAAGGCAGAGTGTGGAGCCTAGCAAGT
TCTCGGACATTATGGACATTAGAACTATGAACTACCACACAGCTCCTTATCAGCCATCGGGATCAATAG
ACATACTGTTCCCCGGCACGTACTACCTCATTGACATTGATGAACTAAGAAGACGTTTTTATGCAAGGA
AAGTTTGA

>Helicoverpa armigera Mevalonate kinase

ATGTTGAACGGAATAGATAATTTGTACACAGTAAGTGTGTCGGCTCCTGGGAAAGTGATTCTTCATG
GGGAGCATTCGGTTGTGTACGGGAAGACTGCGGTGGCTGTCAGTCTTGGACTCAGGAGCTCTATTGT
TATTAAGGAAGTAAATTATCACGTGGAGCCAACGATACGGATACATTTGCCATGCGTCGGATTAGAA
GAAACATTACTTTTAGAACCTACAGTTAAGAGCTTATTCCACCCGAAGCTAGCTCCAGGGATTACGG
GCAAATTCTCTTGGAGATTACCTGATAAGCTAGACCATGACAACCATTTGCGAAGGGTCGACGAGTT
CCTACACATCATTAAGCCAAACTTCGATGCCCTGCAGAATAACCAGAAGAATTCCTTGCGCAGTTTC
CTATATGTGTTTTCTGGGATATTCGGGAGCACGAATCTTCCTGTCCGATCAATGGATATTTCTTTAGG
ATCTGAGTTAACTATTGGAGCTGGTACTGGAAGTTCAGCTTCTTTTGCTGTGTGTTTAGCAGGAGCGT
TAATCCAGCTCCTCAAATTGAAGAGTTCGACATCGTTCGATGCTTTCTATGATCAGACGAGTGAAGA
CTTTAGTATGAATGAAAGAGAAATCATATCGGAATGGGCGTATAATTGTGAGAGGATAATGCACGG
ATCTCCATCAGGTATAGACAACGCAACTTGCACCTTTGGTTCGCTGGCCTCCTTCAAGAAAGGCAGT
AAGCCCCGGCATCTAGACATCAAGATGGACCTCCGGGTTCTCCTAGTGGACTCCCGCGTGTCCCGGG
AGACTCGCGCCCTTGTCGTGAAGGTAGCAGCACTGAGGCAGAGGAACACAGCTGCAGTCGACCATA
TCCTAGACGCTTGTGATCATGTCCCCTCACCCCAGCCACCCCAGGTTTTGGAAAAACTCTCCAGTGG
CAAGTGTGAGATTGATAGTGAAGCGGAGTATCAGCATTTAGCAGAGCTGTGGGACATGAACCACTG
TCTGCTCTCGGCGCTGGGCGTGTCGCACCCCGCTCTAGAAGCAATTCGCAGCGCCGCCCGCGCTAAA
GGACTAGCTTGTAAACTTACCGGCGCCGGTGGTGGGGGTTACGCCATGGTGCTAATCCCGCCGACGA
CGCCGCGGTCCATAGTAGACTCTCTATGCGGGCAACTGCTCGAAAACGGCTTCCGAGTGAAGGAGA
CACGCCTGGGCGGCCCCGGCGTCTCCATCGAGATGTGA
>Helicoverpa armigera Phosphomevalonate kinase
ATGCCTCAAATAATATTGCTATTCAGCGGTAAGAGAAAATCTGGGAAGGACTTTTTAACAACACATTTA
CAGAAATTGCTAGGTGACAAATGCGAAGTGTTAAAAATATCTCAGCCGATAAAGAGTCACTGGGCAA
AGGAGAAAAATTTGAATCTGAATGAGCTGTTGGGTGACGGAGAGTACAAGGAACAATACCGCCTAGA
TATGATCAACTGGAGTGACGAGATGAGATTACAAGATTATGGTTATTTCTGTAGGATCGCTTGTCAGGA
TGCTACAGAGAAGCCAATTTGGATTGTGAGTGATATCAGAAGAAAAACAGACATACGCTGGTTCAAA
GAAAAATATGGGGATCAAATTAGGACTATTAGGATAGTAGCAGATGAAGATACGAGAAAGAAAAGAG
GTTTTCAGTTTAAATCTGGAGTTGACGATGTTGCTTCTGAGTGTAACTTAGATGATTATACTGACTGGG
ATCTGGTTATAGACAATAGTGAAGGAAAACAGGAGGTGGAAGAACAATTGAGTAGTATATTGGGATTG
CTGTCTGGTTTATGA
>Helicoverpa armigera Diphosphomevalonate decarboxylase
ATGAGTGATATTATAACAGTGATAGCACCGGTTAACATAGCTGTAATAAAATATTGGGGCAAAAGGGAC
GAAGATTTAATATTACCGTTAAATGATTCCGTTAGTGCTACTTTAGACACGAGTATTATGTGCGCAAAG
ACATCAGTTTGCGCAAGAGCAGATTTTAAAGAAGATGCAATATGGTTGAATGGGAAGAAGGAATCTTT
TGATAGCCCGAGACTGCAAAACTGTCTTGGAGAAATTAAGTTGCGAGCTGCCGCTGAAAAAAGTGTT
AGTGAAGATTTCTTACAGAGGAAAGTTCATGTGTGTTCTGAAAATAATTTCCCTACTGCAGCTGGGTT
AGCGTCGTCTGCTGCTGGCTATGCCTGTCTCGTGACTGCCCTGGCGAAACTTTACAAGGTAAAGTCTG
ATGTCAGTTCAATTGCTAGGCTGGGCTCAGGCAGTGCTTGTAGGAGTGTATACGGTGGCTTCGTCAGA
TGGCACGCAGGCATCAACCCAAATGGAACAGATTCTGTAGCAACTCAAATTGTTGACTCTACACACTG
GCCTGAAATGCATGCCCTCATACTAGTTGTCGGTGACACTAAAAAGAAAACTAGTTCTACAGTTGGCA
TGAGAATAGCTACTCAAACTTCCGAACTATTAAAACATAGGGTAAAGTATTGTGTGCCGGAGAGAACT
GAGGAGATCATACAGGCAATAAGGCAGAAAGATTTCCCAAAGTTTGCAGAAATTACTATGAAGGACA
GCAATCAGTTCCACGCAGTCGCTTTAGATTCCTATCCTCCATGCGTGTACATGACTGATGTATCTCATTT
CATTGTTGATTTAATTCATAAGTACAATACTGCCTCTGGAAGTGTAAAAGTGGCGTACACATTTGATGC
CGGACCAAATGCTTGCTTGTACCTACTTAAAGATGAGGTACCCAAAGTAATATCTTTGATAAAGCATGT

CTTTCCTACATCAAAGCCAGAAAGCTTTATAACTGGCCTAAGTAGTGGTGAAGAGGTTTTAAGTCCAG
CATTGATGAGTATTTCTGAACCTCAGGTTCAGGATTTGATCAAATATGTGATTCACACAAAAGTAGGTG
AAGGGCCTACAGAAATAACTGATGGGTCACATCTCTTGAATGAGGCTGGATTACCAAAGACTGAATAG

>Helicoverpa armigera isopentenyl-diphosphate delta isomerase 1

ATGCTTGTTCGGAGACTTACAAGATCCTTATGGAACACGGTGAGAGTGGAAAAGAGGTTTCTAGCCTC
TGAACCGCTGAAACCACAGACTGAAGAAAAAGATAGCGTTGACCCAGTACAGTTGGAGGCTCTCGAT
AAAGACATATGTCTGTTAGTTGATGAGAAGGACAACTTCGTGGGAACAGCCACCAAAAGAGAATGCC
ATAAAGTAGGAGATGATGGTGAAGTACTGCTCCACCGTGCCTTCAGTGTGTTTCTCTTCAACAAGAGA
GGAGATATGCTCATGCAGAGAAGGTCCAGCCAAAAAGTAACATACCCAGACTACTACACAAACGCGT
GTTGCAGTCACCCGCTATACATAGATGAGAAAGCCGAAGATGTTGTCACAGCAGCTAGACGCAGGCT
CAATTTGGAGTTAGGAATTCCTTTAGACCAGATGGACCCAGAGTTGTTCACGTTCATGACGCGCGTGC
ACTACCACGACCCCGGCGACGGCGTGTGGGGAGAGCACGAGATCGACCACATCCTATTCTTCCAAGC
AGACGTCAAGGTCAAGCCGAACTCCAACGAGATATCTGAGTATTGCTTCGTACCTAAAGCTGAATTTA
ATGCGTTTATCCCAACTCTGGAAGGTCCACTAACGCCATGGTTCAACATGATTCGCCGACACCGACTC
AAGCTATGGTGGGACAACCTTCATCGTCTCAAGGAATTGGCAGAACCTGACAAGATACAGAAGTTTAT
GAGTGATAAAAAATAA

>Helicoverpa armigera Farnesyl pyrophosphate synthase 1

ATGGCCCTTTTGCTCCGTAAAACATTATTTTTCAACCAAATTAGGGCATTGCATAAAAAAGCGGTTAAA
TTAAGTACACCAATAACTTTCAAATCGGGTGAAAATTTTAAGAAATTTGATGCAGTTCGGCCCGAAGT
GATGAAATCCTTAATGTCTAGTCCTAAGTTCACGAAACAATTGCCTGAAGTTGGTACCAGGATTAAAG
AGATGTTAGATTACACAATGCACGGAGGAAAACGTAGCGTCGGATTATCTGTTCCCTTCGGCTATCAG
ATAATGGAAAATCCAAAGTACTTCGTAGAGGAAAAACTGCACACAGCACGATTTCTAGGATGGTGCTT
AGAAATTTTACAGGCAAACCTACTGACTATAGACGATATATTGGACGCAAACACGACTCGCCGTGGCC
TGACTTGCTGGTACCTGCGACCAGAAGTCGGTACTTCGGCGACTAACGACAGCATGCTCATATATCTAT
GTCTGATGGAAGTCCTGCAAATTAACTTCGAGAAAGAACCTTATTATGTGGATTTGATTAAGATTATTC
ACGATACAGCCATGTACACAGGAATAGGACAATATTTAGAGTACTCATCAAGTTACAGTAAGGAAAAA
AATAATTTGGAAGTATTCAACATGGACCGATTCAATACCATCGTTATACAAAAAGGTGCCTATTCCTTCT
TCACAATGCCCTTTCTCGTATCTCTGCTTTTAGTGAAGAACGGAAAAGAAAGAGATCTTACAGAGCAC
ATCGACATCTGCTTTGAAGTTGGTAAACTTCTACAATATCAGAACGACTTCAAAGACGTCTACTGGGA
CAAGGCTACCTATGGAAAAGATGGCACAGATATTCAAGAAGGTAAACTCTCCTGGATTGCCATAACAG
CGCTGGAGCGCTGCAACGAAGCGCAACGTTCTCTTTTCAAAGAATACTACGGCAGCAGAGACCCTGA
ACATGTTAAGCAAATCAAGCAGCTTTATGGTGAATTGCAAATGGATAAAGTGTACGCTGAGTTCGAAC
ATTCTTTTTATGAAAATATGAAACGGCGAATTCATACTTTGCCAACTGAAGGAGAGATACAATACTTTT
TGCAAATATTAGAGGTGTGCCGACAGAGAGCTTATTAA

>Helicoverpa armigera Farnesyl pyrophosphate synthase 2

ATGAATATTTGTAGAAAATTGACCAACTCCAGCTTGCCTGCTGTTGGGAAAAGGATTGCCAATGTTAC
GGCAAAGGATTTTTTTGACAGTACCTTTCAGAGACTTTATAGTTCCACTAATTCGAAGTTTGATTTGAA
TAAGGAAAGGGTGGAGTTCCATAATGCTCTGCCAGGAGTTATCGATATCCTTGCTAAGCATAAAAAGT
TTCAAGAAATGCCAGAAGCTGATAAATGGATGAGAAATGTCCTTAACGGCAATATAATTGGCGGTAAA
AACATGAGGGGTTTAACGACGGTAATGTCTTACAAAATGATTGAAAAACCAGAGAACATAACGGAGG
AGACCTTAAGATTGGCCAGGACTTTGGGATGGTGCGCTGAAATTTTGCAAGCATACTGCCTCGTGTTA
GACGATATCGCGGACGGCTCCATCACTCGCCGAGGCATGCCTTGCTGGTATAGACGAGAAGACGTCG
GCATAGCAAATGCAGTCAACGACGCCACGCTCATCCACTATTGTCTACTGCAGATATTGCGAGCGAAC
TTCGAGAAATCACCTAATTATGTTAATTACTTTCATAATTTCAACGAGACGCTCTTTTACACAAGTCTCG

GACAATATTTGGATATTATGACAGGGCTAACTAAAAAGAACTACAGTTTATTTACTATGGAACACTACG
ATGCTATAGTCAAACATAAATCAGCCTACTATACGTACAAGCTGCCCATATCTCTGGGATTCATGTTGGC
TAACAGGTATAACGAAGAAACTCATAAAGATGTGGATGATATTTCTATGAAATTGGGGAGACTTTTTCA
AATGCAGGACGATTATATCGACTGTTACGGAGATGAAAATATGACCGGGAAAATGGAAGCGATATCC
AAGAAGGAAAATGTTCATGGTTAGCAGTAAAAGCCCTTCAACACTGCAAGCCTAATCATCGCGCAGT
ATTCACGGTTTGTTACGGAAGTAAAGAGCCGGCACACATAGAACGAATCAAGGTACTTTACAACCAG
CTCAAGATTCCGCAGCTTTACAAAGATGAAGAAAATGAAATCTACAACAAGATTGTTCAAAGAATACA
AGATGTATCTTCAGAATCAGAACGAGAATTGTTCCTACAAGTCTTGCATGATACTTATGGGAGAAAAC
ATTAG
>Helicoverpa armigera Farnesyl pyrophosphate synthase 3
ATGAATAACATAAAATTCCTTCGGTTTATGAGTAGAACACGGACGTTATGTGGCGGCTACAACAAACA
TCCGGGATTTCTTCAACAGTCTTTGAAGAGACATATGCAGACAAGTGTGTCAATATCTCAAATAATGA
AGGAAAAAGAGATTTTTCACGATGCACTGCCAGGTATCATAGACAACCTGCAGGCTAGTTCCAAGATA
TCACAACTCCCTGAAGTGGGCAACTGGGTCAGGAAGGTGCTTGAACACAATTTGACTGGTGGCAAAC
ATTCAAGAGGTATAATCACAATGCTATCATATGAGATGTTAGAAACACCTGAAAAAGTTACTGATGAGA
AACTCAAGCTGGCCAGGGTCCTGGGATGGTGTGTAGAAATGCTACAAGCCTACTTCATAGTAGTAGAC
GATATAATGGACAGATCGACAACACGTCGAGGCATGCCATGCTGGTACCGGATGCCGAACGTTGGTCT
AGGCGCTTTAAACGACTCCATACTCATACATTGTGCGATTATGGAAACGCTACAGACGTACTTTGGTAA
CACGGAACATTATGTAGATGTTGTTCGACTTTTTAATGAGGCACTACTCTACACCTCAATGGGACAACA
CTTGGACTTCACGACAGCCCACCATAAAAAAGACTACAGTCTATTCACAATAGACCGTTACGAGTCTA
TTGTGAAGTACAAGACTAGTTGCTATACCTTCAGGTTACCCGTTCTATTAGGGCTGGTACAGGTTCAAG
ATGTCGATAAGAGAATGTATAACGATGTTGATGATATCTGCTACAAGCTGGGCAGACTATTTCAAATGC
AGGACGATTACATAGACTGTTACGGCGACGAATCAGTCACCGGCAAAGCAGGTACCGACATCCAGGA
AGGTAAGTGCTCCTGGTTAGCTGTCACCGCTTTAAAGCGCTGCAACGATACTCAAAGGGAACTCTTCA
CAAACAACTACGGTAGCGATAAACCAGAACACGTCGACATTATCAAGCGGCTTTACGGTGATTTGAGG
CTACCAGAGATTTACCTACAAGAAGAGACTGAACTACATAATGATATTACTTCGAAAGTGAGAGCGTT
GCCCTCTGAAACCGCTCCTACGTTCTTCTTTAAGCTTATAGACAGGATTTATAAGAGGAAACATTAA
>Helicoverpa armigera Farnesyl pyrophosphate synthase 4
ATGTTCTCCACGAAGAAAAGTTTGGATAGGATCCTACAGATCTACAAGAAAGAGATGCGTCGGCAAAT
CAGCAAGACCACCAGTGTCACCAACTCTGACGCCATGGTGCCAAGGTTGGACCAGGCATCCACCAAG
TCACCTCAGAACGACGAAGCTGGTCCCAAGAAGCTTTTGAAACTGCAAAAATATCACAGATACCTGT
CGACCCTGACAACGCAGGAAATGCCGATGGCTACCCGAGGACTGGCCGTGTCGAAGGACCAGTCCC
GGGAGTTCATGGCTTGCTTTCCGGACATCGTGAGGGACCTCACTGAGACTGGCAAGCACATTGATGT
GCCAGAAGCCAGCAAGTGGTTGGCTAAGCTGTTACAATACAACGTGCCGAACGGTAAAAAGAATCGA
GGTCTAGCGACAGTGCTGGCTTACAAAATGCTGGAAAAGAAAGAAAACTTGACGCCTGAGAATATTC
ACTTGGCCAATATGATGGGCTGGTGCGTTGAAATGTTCCACACTCACCAGTTACTGCAGAATGACATC
GTAGAAGGCACAGAGATGCGTCGCGGCGCCCCCTGCTGGTACAAGCACCCAGAAGTCGGTCTCACTG
GCATCAGCGATGCAGCTCTCGTCCAGGCCGCCATGTTCTCAACCCTCAAACGTCACTTCAACAACAA
ATCGTACTACAGAACCGTCCTTGAAACATTCAATGAGATGCTGTTGAAATGCTCGATCGGCCACTTCC
TAGAAAATCAGATGGCTAAGACGGATAAGCCTGACCTTACCCTGTTCACAATGGAGAAGTACGAAGC
CATCACGAAGTACAAGACTTCATACCACACGTTCCAGATGCCAGTCACCCTGGCTCTGCTCATGACAG
GCGTAGAGGACCCCGAAACCCATAGACAAGCCAAAACAATCCTCCTTAAGATGGGCGAATTCTTCCA
AATTCAAGATGACTTCTTAGATTGCTTCGGAGATCCAGCAGTCACAGGCAAGAACGGCACTGACATTC
AAGATGGCAAATGCACTTGGCTCGCAGTTGTCGCCTTACAGAGGGCAACACCGAAGCAGAGGCAATT

CATGGAAGAGAACTATGGCAGCTCAGACCCTGAAGCAGTCGCCAAGATCAGGCACTTGTACGAGGA
ACTGCAGCTGCCTCACACATACTCTGTCTTCGAAGACGCTACTTACGATCTCCTTAGGACTCAAATCC
AGCAAGTCACAAGAGGCCTGCCCCACGAACTGTTCTTCAAAATCTTAGACAACATCTTCAGGCGAAG
CGTCTAA
